# Data-driven human transcriptomic modules determined by independent component analysis

**DOI:** 10.1186/s12859-018-2338-4

**Published:** 2018-09-17

**Authors:** Weizhuang Zhou, Russ B. Altman

**Affiliations:** 1 Department of Bioengineering, Stanford University, Stanford, CA 94305 USA; 2 Department of Genetics, Stanford University, Stanford, CA 94305 USA

**Keywords:** Independent component analysis, Gene expression, Functional modules, Transcriptome

## Abstract

**Background:**

Analyzing the human transcriptome is crucial in advancing precision medicine, and the plethora of over half a million human microarray samples in the Gene Expression Omnibus (GEO) has enabled us to better characterize biological processes at the molecular level. However, transcriptomic analysis is challenging because the data is inherently noisy and high-dimensional. Gene set analysis is currently widely used to alleviate the issue of high dimensionality, but the user-defined choice of gene sets can introduce biasness in results.

In this paper, we advocate the use of a fixed set of transcriptomic modules for such analysis. We apply independent component analysis to the large collection of microarray data in GEO in order to discover reproducible transcriptomic modules that can be used as features for machine learning. We evaluate the usability of these modules across six studies, and demonstrate (1) their usage as features for sample classification, and also their robustness in dealing with small training sets, (2) their regularization of data when clustering samples and (3) the biological relevancy of differentially expressed features.

**Results:**

We identified 139 reproducible transcriptomic modules, which we term fundamental components (FCs). In studies with less than 50 samples, FC-space classification model outperformed their gene-space counterparts, with higher sensitivity (*p* < 0.01). The models also had higher accuracy and negative predictive value (*p* < 0.01) for small data sets (less than 30 samples). Additionally, we observed a reduction in batch effects when data is clustered in the FC-space. Finally, we found that differentially expressed FCs mapped to GO terms that were also identified via traditional gene-based approaches.

**Conclusions:**

The 139 FCs provide biologically-relevant summarization of transcriptomic data, and their performance in low sample settings suggest that they should be employed in such studies in order to harness the data efficiently.

**Electronic supplementary material:**

The online version of this article (10.1186/s12859-018-2338-4) contains supplementary material, which is available to authorized users.

## Background

The human transcriptome, a snapshot of all mRNA molecules in a cell or tissue, is invaluable in advancing precision medicine. Many public databases have been established to map drug responses to transcriptomic profiles, such as the Welcome Trust Sanger Institute’s Cancer Genome Project (CGP), the Connectivity Map (CMap) [1] and the Library of Network-based Cellular Signatures (LINCS). While the ability to measure gene expression levels of nearly every expressed gene in a cell allows for precise characterization of tissues at the molecular level, transcriptomic data is inherently noisy due to the dynamic nature of transcription. This makes it difficult to identify patient subtypes when the effect size is small, and also confounds direct interpretation of analysis results. Statistical methods to handle such high-dimension data typically control the false discovery rates through *p*-value corrections and q-value thresholding, or increase power via the simultaneous study of multiple genes (i.e. gene sets). Gene set enrichment analysis (GSEA) [2, 3] is widely used today in transcriptomic analysis, and is facilitated by the Molecular Signatures Database (MSigDB) [3], a database with 17,779 gene signatures across seven collections. In practice, researchers running GSEA typically choose a particular subset or collection from MSigDB based on what they believe to be related to the tissue or condition of the sample. This may induce biasness in the analysis as GSEA is sensitive to the choice of subsets used [4], and also the amount of gene filtering steps done during preprocessing [5]. Furthermore, Liberzon et al. [6] also found significant redundancies in MSigDB’s signatures, which can skew the reported enrichment scores from GSEA.

An alternative to using user-defined gene sets is to employ data-driven approaches to construct lower-dimension features so that the statistical power can be increased. Principal component analysis has previously been performed on microarray data to summarize the experimental dataset containing tens of thousands of genes to a feature space that is a hundred-fold smaller [7, 8]. It was observed that while the first three or four principal components can sufficiently capture most biological signals in a large microarray dataset [9–11], they fail to do so when there is a small effect size and/or when there is a small number of samples exhibiting the effect [12]. Recent analysis performed by Tan et al. on *Pseudomonas aeruginosa* gene expression profiling experiments [13, 14] showed that components derived from PCA had fewer associated biological pathways than components from competing methods such as ICA. More fundamentally, the underlying assumption of PCA (that the data is a multivariate Gaussian) does not hold for transcriptomic data, which are typically super-Gaussian. Lee and Batzoglou [15] suggested the use of a related technique, independent component analysis (ICA), as a more faithful model for such non-Gaussian data. The statistically independent components obtained from ICA have been reported to have biological significance [16–18], and are alternatively known as metagenes, transcriptomic modules or functional components (FCs). Unlike gene sets, where a gene’s membership is binary, functional components present a smoothed and continuous version of set membership, better reflecting the complex network and co-dependency of genes. A sample transcriptome can then be expressed as a linear combination of these functional components:

$$ {g}_i={\sum}_{f=1}^n{w}_f{F}_{fi}+{\epsilon}_i $$

Where *g*_*i*_ is the expression level of gene *i*, *w* is the coefficient of the corresponding functional component *F*, and *ϵ* is the noise in the measurement.

The extensive corpus of public available microarray data is useful for identifying functional components that are representative of fundamental human biology. These “data-derived” features have the advantage of not being dependent on expert prior knowledge, and can be used across different experimental conditions. In particular, analysis pipelines built on these features do not require user-defined parameters, thus increasing reproducibility of results. Engreitz et al. [19] previously demonstrated the use of ICA to identify such features based on a set of 9395 microarrays from GEO, but the methodology employed resulted in many correlated components, with the maximum correlation being 0.802. We have leveraged the exponential growth of GEO over the past decade to obtain a ten-fold increase in data for training our ICA model. Additionally, we have chosen to use the ICA algorithm *ProDenICA* [20], which has been previously documented to have higher sensitivity to a wider range of source distribution and better general performance than the more common *FastICA* algorithm [21]. Although the original authors of ProDenICA demonstrated its usage in relatively small datasets, the method has been extended to larger datasets recently, most notably by Risk et al. [22] in their application to large fMRI data. In this paper, we apply the algorithm to an even larger dataset based on the human transcriptome (20,089 genes), and identified a set of 139 functional components from a diverse range of human microarray data. Using six different studies from GEO, we demonstrate the usage of FCs in transcriptomic analysis. Specifically, we present the following:

1. Rigorous quantification of the 139 FCs through multiple repeats and subsampling of data to ensure reproducibility of the components. We also constructed a tissue fingerprint library based on GSE3526 and GSE7307 so that query samples can be quickly mapped to the most similar tissue.
2. Demonstration of the FCs as machine learning features for sample classification in two different studies from GEO (rheumatoid arthritis, GSE71370; leukemia, GSE13159). We show that the FCs can be used as classifier features without prior processing, as opposed to typical workflows that require the identification of DE gene sets before model training. We also evaluated their robustness in dealing with small training sets by subsampling the data from GSE13159 at different sizes. The performance of the models built using the FCs was then compared to the ones built using the original genes, and found to be superior when sample sizes were small. We note that this makes our methodology particularly useful for typical studies where the training set consist of less than 50 samples.
3. Demonstration of FC’s ability to regularize data, using data from the MicroArray Quality Control (MAQC) study and a multi-center AML study, GSE15434. The FC-space clustering of MAQC samples is comparable to that of the original gene-space, and analysis of the AML study in FC space also produces more parsimonious results across the different centers.
4. Evaluation of biological relevancy of differentially expressed FCs. We apply differential expression analysis to two different studies (rhabdomyosarcoma, GSE66533; dengue virus infection, E-MTAB-3162) and show that the significant FCs in both cases had biological annotations that were similar to the results from the original papers based on gene-level analysis.

## Methods

### Data collection

Raw data was collected as in [23]. Briefly, we obtained all human GEO series records (GSEs) that were found on the Affymetrix HG-U133 Plus 2.0 platform (GPL570) as of March 2015. After filtering for GSMs with associated raw CEL files, we obtained 2753 GSEs, containing 97,049 microarray CEL files. The CEL files were then processed using robust multi-array average (RMA) [24, 25] and corrected for technical bias [26]. The probes were then mapped to 20,089 unique Entrez gene identifiers using the R package *Jetset* v3.1.2 [27].

The dataset, containing 20,089 genes by 97,049 arrays, was then quantile-normalized between arrays, and gene-centered. This was followed by scaling and centering of the dataset by array. We denote the resulting matrix by *F*.

### Constructing a representative compendium

The Spearman’s rank correlation coefficient (*ρ*_*i*, *j*_) was computed between all arrays. Distances between arrays *F*_*i*_ and *F*_*j*_ were defined as 1 – *ρ*_*i*, *j*_, and hierarchical clustering of the arrays was performed using average linkage. The maximum intra-cluster distance (cutoff height of tree) was determined by using a k-nearest neighbor knee plot, and the tree was cut accordingly to obtain the corresponding clusters. We excluded clusters with less than five members and selected the medoids of the remaining clusters as representative samples. We refer to the collective set of medoids as the representative compendium, and denote it by *X*. To characterize the samples in the representative compendium, we extracted the corresponding metadata (title, source name, characteristics, description and treatment protocol) from GEO using *GEOmetadb* [28] and then parsing them with BioPortal’s Annotator [29] to get the associated NLM’s Medical Subject Headings (MeSH) descriptors. We mapped the descriptors to their highest level term, and retained only the terms from the following four categories: A (anatomical terms), C (diseases), D (drugs and chemicals) and G (phenomena and processes).

To determine the relationship between the size of a derived compendia and the time, we repeated the process across various calendar years in the GEO repository. Arrays in GEO at the end of each calendar year were processed similarly to the above to yield both the full compendium and the corresponding representative compendium for that year. The number of arrays in both compendia was then tabulated as a function of time.

### Whitening and selection of number of components

Whitening (decorrelation of variables followed by scaling) of the data matrix [30] was done using singular value decomposition (SVD), *X* = *UDV*^*T*^. The orthogonal matrix U is then inputted to the ICA algorithm. The diagonal values (*d*_*ii*_) of the diagonal matrix D is related to the eigenvalues (*e*_*i*_) of the covariance matrix (*X*^*T*^*X*) by the transformation ${e}_i=\frac{{d_{ii}}^2}{g-1}=\frac{{d_{ii}}^2}{20088}$ . The correction of *g* − 1 is necessary for consistency with the unbiased estimate of variance.

The eigenvalues of the covariance matrix provides a way to select the number of components. In particular, parallel analysis is a well-documented method to stably perform the selection [31–34]. We performed 5000 simulations by running SVD on random matrices of the same dimension as the input matrix *X*. For each sequential component in the simulations, we obtained the median (Horn’s method [31]) and 95-percentile (Glorfeld’s method [32]) of the corresponding eigenvalues across the simulations, and used them as the bias. We then subtracted the bias from the actual eigenvalues of *X*, and retained the components (*n*) whose corrected eigenvalues were greater than 1. We define the whitened and reduced data matrix *Y* (*g* × *n*) as

$$ Y=\sqrt{g-1}\times U $$

$$ X= UD{V}^T=\frac{1}{\sqrt{g-1}}\times YD{V}^T $$

Where *g* = 20089 genes, and the square root term is introduced as a scaling factor so that the resulting diagonal matrix from the SVD of *X*^*T*^*X* would be directly comparable to eigenvalues of the covariance matrix. We used the data matrix *Y* as input for ICA.

### Independent component analysis

We implemented ICA using the R package ProDenICA [21]. The convergence threshold was set to 1e-6, with a maximum iteration of 8000. Additionally, we set the number of grid points for density estimation to be 2000, and the robustness parameter (“order”) to 11. ICA produces the following output:

$$ Y= SA $$

Where the source matrix *S* has dimensions *g* × *n*, and the mixing matrix *A* has dimensions *n* × *n*.

A total of 100 independent runs of ICA were performed on the input data *Y*, and the solutions were processed in a similar method to Risk et al. [22]. First, all solutions were converted to their canonical form by ordering the ICs (columns of *S*) by their respective skewness. The solution with the highest negentropy score across the 100 repeats was chosen to be the “best solution”, and was then compared to the other 99 runs component-wise. For the source matrix from the k-th run, *S*^*k*^, we computed the pairwise-component Pearson correlations with the “best solution”, *S*^0^. We define the cost matrix *ℂ* to maximize these correlations:

$$ {r}^{-}=\frac{1}{2}\left(1-\rho \right) $$

$$ {r}^{+}=\frac{1}{2}\left(1+\rho \right) $$

$$ \mathbb{C}=\min \left({r}^{+},{r}^{-}\right) $$

Where *ρ* is the Pearson correlation matrix. Minimization of the overall cost is a linear assignment problem, and was solved using the Hungarian algorithm (R package *clue* [35]). Let ***B*** be the matrix that represents this assignment, such that ***B***_***i,j***_ = 1 if the i-th component of *S*^0^ was assigned to the j-th component of *S*^*k*^, and zero otherwise. The elements of the signed permutation matrix ***P*** is then defined as

$$ {\boldsymbol{P}}_{i,j}=\left\{\begin{array}{c}1,\kern0.5em \mathbf{1}\left[{r}_{i,j}^{-}<{r}_{i,j}^{+}\right]\\ {}-1,\kern1.5em \mathbf{1}\left[{r}_{i,j}^{-}\ge {r}_{i,j}^{+}\right]\end{array}\right. $$

For ***B***_***i,j***_ ≠ 0, and zero otherwise. The permutation matrix is a 1–1 mapping and rearranges the columns of *S*^*k*^ (with appropriate reorientation of direction) so that the correlations with the respective columns in *S*^0^ are maximized. The component-wise correlations of the 99 solutions with the “best solution” is then

$$ {c}_i^k= cor\left({S}_i^0,{\left[{S}^k\boldsymbol{P}\right]}_i\right) $$

Where ${S}_i^0$ and [*S*^*k*^***P***]_*i*_ are the i-th component (columns) of the “best solution” and the permuted source matrix from the k-th run respectively.

### Evaluation of component estimates

We resampled the full compendium randomly without replacement to obtain 50 similar-sized pseudo-representative compendiums. Whitening was performed as described previously, but we selected the same number of components as the original solution to facilitate comparison between the models. For each of the 50 resampled compendiums, we ran ICA ten times, and chose the solution with the highest negentropy score as the solution for that resampled compendium. We compared the 50 chosen solutions to the “best solution” from the representative compendium using the same methodology as per the previous section.

### Biological annotations of components

#### GO terms and relationship to the H collection in MSigDB

For each component, we defined the sets of genes with loadings that were three standard deviations above or below the mean as the up or down modules respectively for the component. Collectively, we term the union of both set of genes as active genes for the component. As per Engreitz et al. [19], we performed GO enrichment analysis, using TopGO [36] on the up and down modules separately.

The percentage overlap between gene signatures from the H collection of MSigDB [3] and the active genes for each FC was calculated. For each gene signature-FC pair, we also checked for enrichment of overlapped genes by performing a hypergeometric test; only pairs that had a BH-corrected *p*-value of less than 0.01 were retained.

#### Fingerprinting human tissues: GSE3526 and GSE7307

All 353 normal human samples from GSE3526, coming from 65 different tissue types derived from ten post-mortem donors, were downloaded from GEO, processed and projected into FC space. To obtain representative samples from the 22 nervous system tissues, we calculated the pairwise distances within each tissue type and selected the medoid (sample with the minimum distance to all other samples within the same tissue type). Clustering of the 22 samples was then performed. The set of all samples from GSE3526 were also used as a compendium to annotate queries with their most similar tissue origin.

GSE7307 (Human Body Index) contains 677 samples from 90 tissue types, some of which were from diseased patients. We downloaded only the healthy samples, and processed them as per GSE3526. We compared the tissues types that were common to both GSE3526 and GSE7307 using Pearson correlation coefficient. To provide robust estimates that were not affected by outlying samples in the tissue types, we reported the median and the standard deviation of the correlations for each “GSE/tissue”-“GSE/tissue” pair. We also included the sub-compendium (“Human Tissue Compendium”), containing both 353 samples from GSE3526 and 504 samples from GSE7307, in our R package so that users can also use it to annotate their query samples with the most probable tissue types.

### FC applications and analysis

For all evaluation datasets, the raw CEL files were downloaded from GEO. Each GSE was processed independently by running RMA on the set of samples, followed by technical bias correction as per the earlier section “Data Collection”. Projection of a dataset *Q*_*gene*_ into the corresponding FC space is done via the matrix multiplication

$$ {Q}_{FC}={S}^T{Q}_{gene} $$

A unitary vector space based on the FCs loadings can also be defined by normalizing each component to have unit length, which we provide as an option in our R package. For all analysis in this paper however, projection onto FC space was done using the original gene loadings in the calculated FCs.

Wherever t-test was used, Benjamini-Hochberg correction was performed on the *p*-values [37], with N either being the total number of FCs (139), or the total number of genes (20,089) being tested. For heatmaps, the genes and arrays were clustered using hierarchical clustering with average linkage, and the distance metric for both was defined using the Pearson correlation:

$$ Dis{t}_{i,j}=1- cor\left({Q}_i,{Q}_j\right) $$

#### FCs as features for machine learning algorithms: GSE71370 and GSE13159

For GSE71370, the meta-data available in GEO was used to annotate the samples under three categories: synovial fluid from rheumatoid arthritis (RA) patients (RASFM), peripheral blood from RA patients (RAPBM) and peripheral blood from healthy patients (HCPBM). Gene expression data were projected into FC space. For each of the three pair-wise comparisons between categories, unpaired t-tests were performed across the FCs, with a BH-corrected *p*-value threshold of 0.05. The union of the three sets of differentially expressed FCs was then used as the signature to cluster the sample types. We performed hierarchical clustering based on the FC values in the signature, using average linkage. To identify FCs that were specific to the differences between RAPBM and RASFM, we focused on the DE FCs that were unique to the pair (I.e. not in common with DE FCs from the RASFM vs RAPBM analysis), and report the corresponding GO enrichment annotations.

GSE13159 contains data from the Microarray Innovations in Leukemia (MILE) study program, consisting of eighteen different categories of leukemia (including a control group). The class labels of the individual sample were obtained from the Data Supplement accompanying the original publication [38]. After preprocessing as described earlier, the data was projected into FC space using the unitary vector space. The classification results were obtained using the same methodology of the original authors, by applying support vector machine (SVM) classifiers in three independent runs using 30-fold cross validations. The R package *kernlab* [39] was used to implement the classifiers with a linear kernel function. We also defined the call rate (CR) similarly as the number of determinable calls. The sensitivity for each class was calculated as the fraction of correctly predicted samples in that class out of all determinable calls in the run. We report the mean CR and sensitivity across the three runs.

#### Performance of FC-based models in low sample settings

Samples from classes C3 (c-ALL/pre-B-ALL with t(9;22), 122 samples) and C8 (c-ALL/pre-B-ALL without t(9;22), 237 samples) in GSE13159 were defined as the positive and negative groups respectively. For a given simulation run, we randomly chose 22 C3 and 37 C8 samples as the held-out test set. The remaining data in the two groups (100 C3 and 200 C8) were then subsampled at 5 10%, 20%, 40%, 60% and 80% to produce corresponding training sets for training SVM classifiers (same parameters as the above analysis for GSE13159). For each subsampling percentage, we repeated the sampling 200 times. For a particular sampling, we calculate the negative predictive value (NPV), positive predictive value (PPV, aka precision), sensitivity (aka recall), specificity and accuracy as follows:

$$ P=\left[ Class=C3\right];N=\left[ Class=C8\right] $$

$$ Pred.P=\left[ Predicted\ Class=C3\right]; Pred.N=\left[ Predicted\ Class=C8\right] $$

$$ {TP}_x=\#\left[P\cap Pred.{P}_x\right];{TN}_x=\#\left[N\cap Pred.{N}_x\right] $$

$$ {PPV}_x=\frac{TP_x}{\#\left[ Pred.{P}_x\right]} $$

$$ {NPV}_x=\frac{TN_x}{\#\left[ Pred.{N}_x\right]} $$

$$ {Sensitivity}_x=\frac{TP_x}{\#\left[P\right]} $$

$$ {Specificity}_x=\frac{TN_x}{\#\left[N\right]} $$

$$ {Accuracy}_x=\frac{TP_x+{TN}_x}{\#\left[P\right]+\#\left[N\right]} $$

Where x is the model based on feature under consideration (FCs or genes).

In addition, for each sampling, we also performed McNemar's test with continuity correction on the classification results from the two models (FC vs gene space). If the calculated test statistic for the pair of models had a *p*-value that was less than 0.05, we defined them to be significantly different. When there is perfect agreement between the pair, the test statistic is undefined; in this case, we simply note that the two models are identical. For a given run, we compute the percentage of the 200 samplings where the two models were observed to be different.

The average across the 200 sampling for a given subsampling percentage were then recorded as the respective statistic for that run. A total of ten independent simulation runs were performed, and the mean and standard deviation for the statistics were reported across the ten runs. For comparison, we also calculated the above statistics using the full remaining data (i.e. subsampling percentage is 100%) at each run. Repeats were not performed for this case.

#### FCs retain biological information while regularizing data: MAQC and GSE15434

Affymetrix HGU-133 Plus 2.0 samples from the MicroArray Quality Control (MAQC) project were downloaded from GEO (GSE5350) and processed. The 120 samples were clustered in both FC space and full gene space, and cophenetic correlation between the trees was computed. For visualization purposes, a tanglegram [40] using both trees was also generated. For evaluation of the FC-based clustering tree, we grouped samples from A and C as a mega-class, and B and D as the other mega-class. The tree was cut to yield two clusters, and these were then classified as one of the two mega-classes based on the majority of the cluster membership. The purity of the clustering was calculated as

$$ Purity=\frac{1}{120}\sum \limits_{i=1}^2\# Correctly\ Classified\ Samples\ in\ {C}_i $$

Where *C*_*i*_ is the i-th cluster. The Gini impurity for each of the two clusters was calculated as

$$ Gini\ Impurity\left({C}_i\right)=1-\sum \limits_{j=1}^2{f}_j^2 $$

Where *f*_*j*_ is the fraction of samples in the i-th cluster that are from the j-th mega-class.

GSE15434 contains a total of 251 AML samples, coming from three different centers in Germany: Dresden (DRE), Munich (MUC) and Ulm (ULM), with 78, 96 and 77 samples respectively. Approximately half of the samples contained mutations in the NPM1 gene. We identified differentially expressed (DE) functional components (FCs) and genes between the NPM1-mutated and NPM1-wild type groups using the R package *limma* [41] at a false discovery rate threshold of 1%, and compared the number of shared DE FCs/genes between the three test centers. We also performed a typical gene set enrichment analysis [3] using the 4725 curated gene sets in the C2 collection of MSigDB v5.0, using the recommended parameters of 1000 phenotype permutations and a false discovery rate (FDR) of 25%. Significant gene sets were identified for both NPM1-mutated and NPM1-wild type groups.

#### Differentially expressed FCs are biologically relevant: GSE66533 and E-MTAB-3162

For GSE66533, the rhabdomyosarcoma samples were separated into two main groups (33 PAX3-FOX01 Fusion-Positive and 25 Fusion-Negative samples) based on descriptions obtained from Supplementary 1 of the paper by Sun et al. [42]. Gene expression data were projected into FC space, and unpaired t-tests were performed across the FCs to identify DE FCs. To perform a search for similar samples, we calculated the Pearson correlation coefficient in FC space between samples from the study and the full compendium. For each sample in either group, we retained all GSMs from the full compendium that had a correlation of more than 0.95, and term these “neighbors”. We then took the union of these “neighbors” within a group, and removed GSMs that were not considered “neighbors” to at least half of the group’s members. To identify GSMs that were unique to either group, we focus on the set-difference between the two sets of “neighbors”. We also applied our “Human Tissue Compendium” to identify the tissue types most closely associated with the samples.

For E-MTAB-3162, the raw CEL files was downloaded from ArrayExpress [43] and processed. The meta-data obtained from the sdrf file, and used to divide the dengue patient samples into the two subgroups (Day 0 vs Day 4). We performed t-test to identify the set of DE FCs. To map the GO annotations to GO slim terms, we used the Map2Slim tool [44] from the Gene Ontology project, with the *go-basic* ontology and the default *goslim_generic* subset.

## Results

### Representative compendium and parallel analysis

**Fig. 1:**
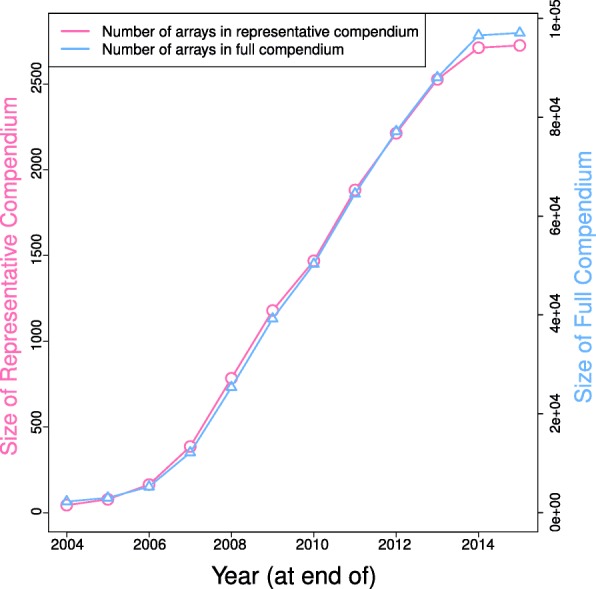
Sizes of full and representative compendium as a function of time. The number of arrays in both compendia is plotted here, as a function of the year. In 2015, there were 97,049 arrays in the full compendium and 2726 in the representative compendium

To avoid overrepresentation of any biological phenotype in the training data, clustering of microarray samples was performed on the full compendium (97,049 arrays) to obtain a representative compendium. The height cutoff of the clustering tree was determined to be 0.3 based on k-nearest neighbor plots for k = 4 and 5 (see Additional file 1: Figure S1). After the filtering step described in the methods section, we obtained a representative compendium consisting of 2726 samples. The clustering and filtering process was found to be robust against varying sizes of the full compendium, and scaled closely with the latter (Fig. 1). 86.4% of the samples have between two to nine unique MeSH terms coming from the four MeSH categories (Additional file 1: Table S1). The MeSH annotations of the representative compendium (Additional file 1: Table S1) indicate that about a third of the samples were cancer-related (MeSH term: C04, neoplasms), with substantial number of representatives from other pathological conditions and diseases found in skin and immune system. There are also representatives from all major anatomical classes (MeSH terms: A0-A9).

**Fig. 2:**
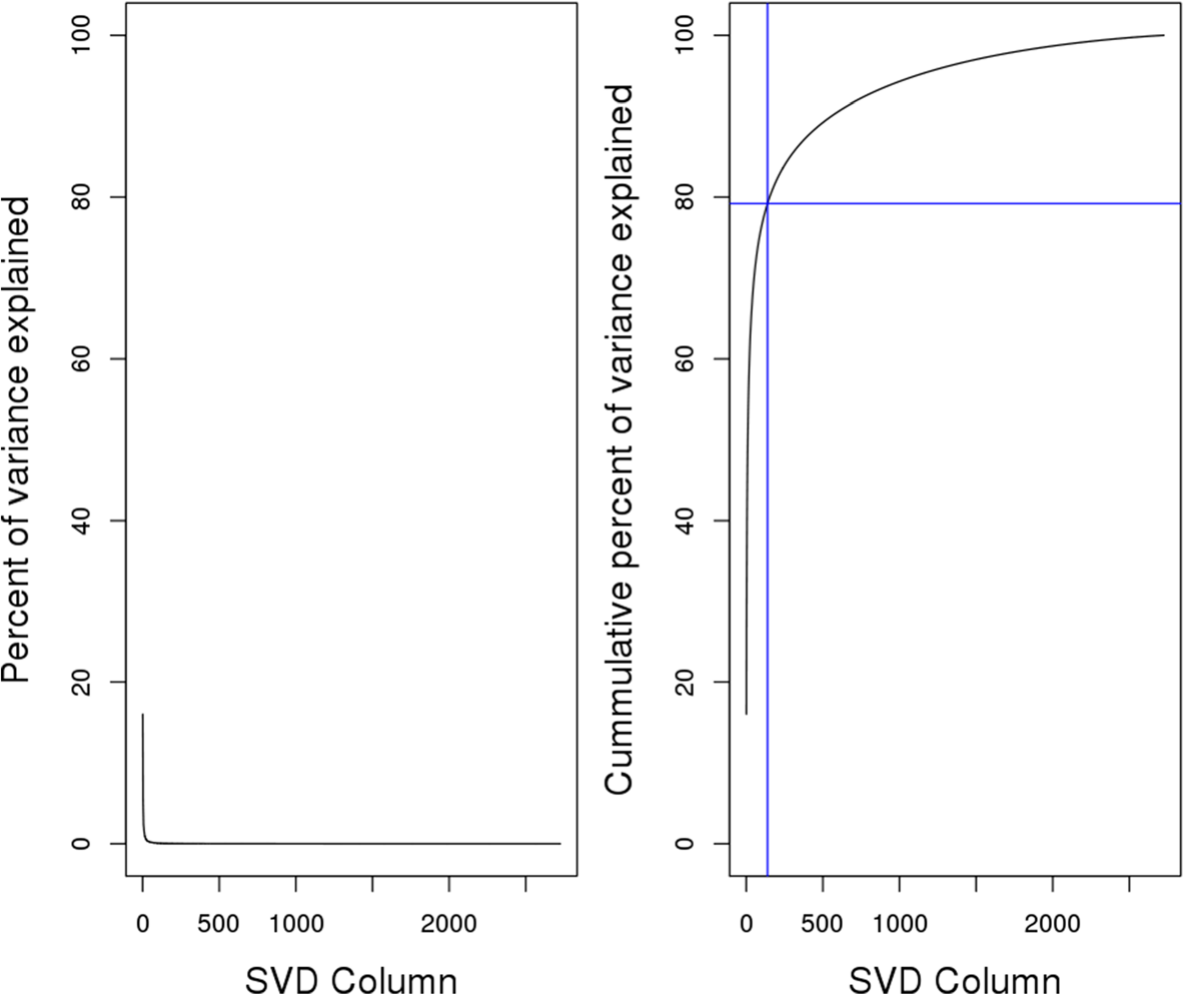
Variance explained by eigenvectors from SVD. (Left) Percentage of variance explained by each eigenvector. (Right) Cumulative variance explained by the eigenvectors. The blue lines represent the cumulative variance explained by the leading 139 eigenvectors

After whitening of the representative compendium, parallel analysis suggested that only the leading 139 components should be retained. We note that the number of retained components was the same for both implementations of parallel analysis, using either the median (Horn’s method [31]) or the 95-th percentile (Glorfeld’s method [32]) for determining bias. Collectively, the 139 components of the whitened data explained close to 80% of the total variance in the representative compendium (Fig. 2). This whitened and reduced matrix (20,089 × 139) was then used for subsequent ICA processing.

### ICA and evaluation of component estimates

On average, each of the 100 independent runs took 2461 iterations to reach the convergence requirement. The final negentropy of the ICA solutions ranged from 0.2417 to 0.2443, with a median of 0.2442. Run 39’s solution yielded the highest negentropy and was thus used as the “best solution” in the rest of the paper. The 139 columns of the canonized S matrix are the independent components obtained from ICA, and we refer to them as functional components (FCs). Each FC had zero mean and unit standard deviation.

**Fig. 3:**
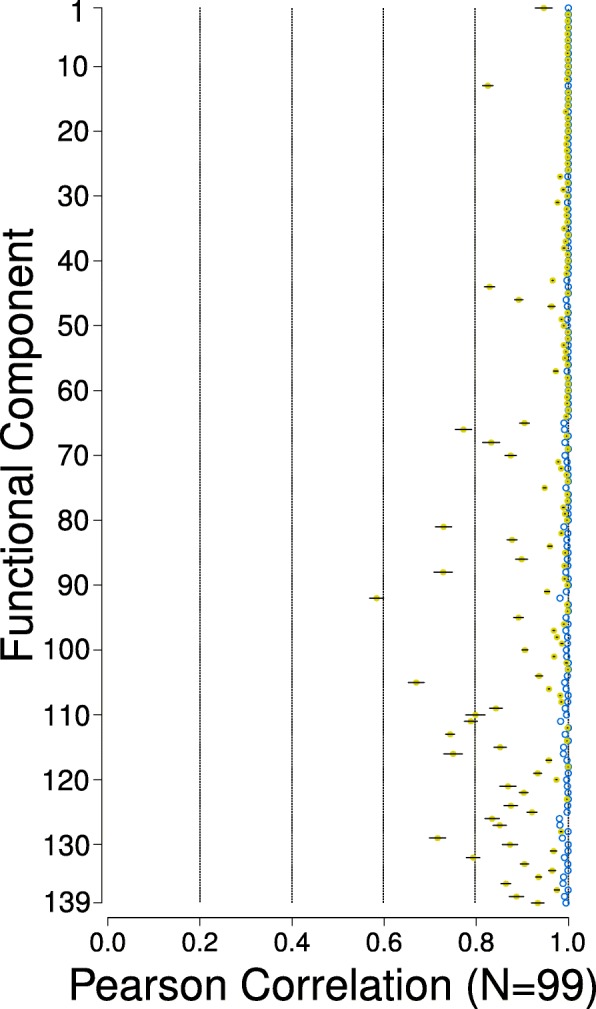
Correlation of FCs between the best solution and each of the 99 other runs. The yellow dots and the black lines are the mean and standard error of the mean Pearson correlation coefficient values respectively, for each FC. The empty blue circles are the maximum coefficient recorded for the FCs

The derived FCs were well correlated between all 100 runs (Fig. 3), and for the majority of the FCs, the mean Pearson correlation coefficient was more than 0.8, with the maximum being close to 1 for all the FCs. Similar results were observed when Spearman correlation was used. We note that the leading 25 FCs of our chosen solution were also highly reproducible in the compendium subsampling analysis, with median Pearson correlation coefficients of more than 0.8. However, the correlation coefficients yielded by the subsampling analysis were uniformly lower than the ones observed in Fig. 3 across the FCs, and greater so for the tailing FCs. In particular, FCs 65 to 139 had a maximum Pearson correlation coefficient of less than 0.8 in the subsampling analysis.

### Biological interpretation of FCs

**Fig. 4:**
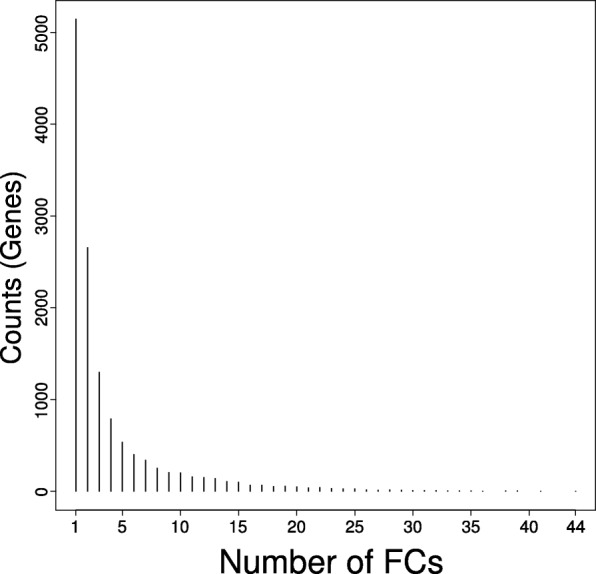
Promiscuity of significant genes in FCs. Significant genes for each FC were pooled together and tabulated. The histogram shows the distribution of how frequently a gene is found to be significant in one or more FCs. The x-axis is the number of FCs in which a particular gene is found to be significant, and the y-axis is the number of unique genes that meets that corresponding requirement. For instance, the maximum number of FCs that a gene was found to be significant in was 44, with only one gene achieving that criteria (AKR1C3; Entrez ID 8644). This observed in the histogram at the 44th position on the x-axis, with a height corresponding to 1 (represented as a dot due to the scale). A total of 9091 genes were found to be significant in only one to three FCs

To gain better understanding of the FCs, we identified the key gene contributors to each of them. The elements of each component are the gene loadings, and can be interpreted as the level of contribution of a gene to the component’s score. For a given FC, we consider the set of genes whose absolute loading is three standard deviations above the mean as active genes. Apart from FC 1, which only had 28 active genes, the number of active genes in the other FCs ranged from 103 to 494, with a median of 382. Amongst the 20,089 genes, 12,978 genes were found to be active in at least one FC. The majority of the genes were active in only up to three FCs (Fig. 4), and the maximum number of components that a gene was observed to be active in was 44.

The active genes for each FC were then used to obtain GO annotations for the corresponding FC. Of the 139 FCs, 22 did not have any GO annotations, and a further 14 had only one GO annotation. The largest number of GO annotation belonging to an FC was 58 (FCs 3 and 4). A total of 689 unique GO codes were obtained across the 139 FCs, a 66% increase compared to the 415 unique GO codes obtained from the corresponding 139 leading principal components. This suggests that there is more biological signal in the FCs than components obtained via PCA, in line with current literature [13]. The GO annotations for some of the FCs are presented in this paper as part of the reanalysis of other gene expression studies; the complete set of GO annotation for the FCs can be found in our R package, *humanFC*.

**Fig. 5:**
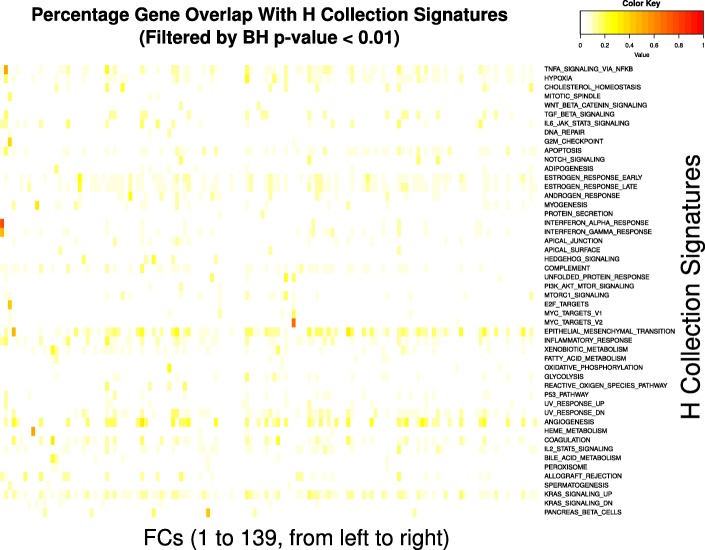
Relationship between FCs and H collection from MSigDB. Overlaps between the active genes and gene signatures from the H collection were filtered for statistical significance and then presented as a percentage of the total number of genes in each signature

The percentage gene overlap between active genes in the FCs and the respective gene signatures in the H collection of MSigDB were calculated, and only the statistically significant pairs are shown in Fig. 5. The highest overlap (75%) occurs between FC 2 and the H collection signature “INTERFERON_ALPHA_RESPONSE”, which contains 97 genes. Half of the signatures in the H collection contain 200 genes each, so even a pair with 50% gene overlap in Fig. 5 can indicate up to 100 shared genes. For instance, FC 10 and the gene signature “HEME_METABOLISM” have only a 52.5% overlap, but the actual number of shared genes is 105. In particular, FC 10 has five GO annotations (GO:0006782, GO:0051597, GO:0015701, GO:0006879 and GO:0048821) that are all related to heme metabolism, supporting a strong relationship with the namesake gene signature.

Twenty of our FCs do not have any significant gene overlap with the signatures in the H collection. Of these, fourteen of them (FC 1, 36, 51, 59, 60, 90, 94, 99, 102, 103, 112, 118, 123, 128 and 137) also do not have any GO annotations. The lack of GO annotations for these FCs does not necessarily indicate a lack of biological significance; for instance, the active genes in FC 1 are clearly markers for sex-specific features (Table 1). Insight into the characteristics of these FCs can also be obtained by looking at the tissue samples microarray experiments that have the highest or lowest score in those FCs. In the case of FC 36, the ten lowest scoring samples were mostly from myeloma cells, whereas the highest scoring samples were from normal epithelia.

**Table 1:** Active Genes for FC 1

Direction	Gene Symbol	Chromosome Number
Down (negative loadings)	EIF1AX	X
	DDX3X	X
	PUDP	X
	KDM6A	X
	PRKX	X
	XIST	X
	NLRP2	19
	TXLNG	X
	ZFX	X
	TSIX	X
	KDM5C	X
	LOC102724689	2
Up (positive loadings)	RPS4Y1	Y
	EIF1AY	Y
	DDX3Y	Y
	ANOS1	X
	PRKY	Y
	KDM5D	Y
	TTTY14	Y
	NLGN4Y	Y
	UTY	Y
	TTTY15	Y
	DDX43	6
	USP9Y	Y
	SPESP1	15
	ZFY	Y
	TXLNGY	Y
	FRG1CP	20

Gene symbols and chromosome number for the 28 active genes in FC 1, grouped by their direction (sign of loadings)

### Fingerprinting human tissues

We built a database of tissue fingerprints so that it could be used to annotate future samples. In order to avoid fitting to errors from a single study, we compared the fingerprints from two relevant tissue studies (GSE 3526 and GSE7307) with each other.

**Fig. 6:**
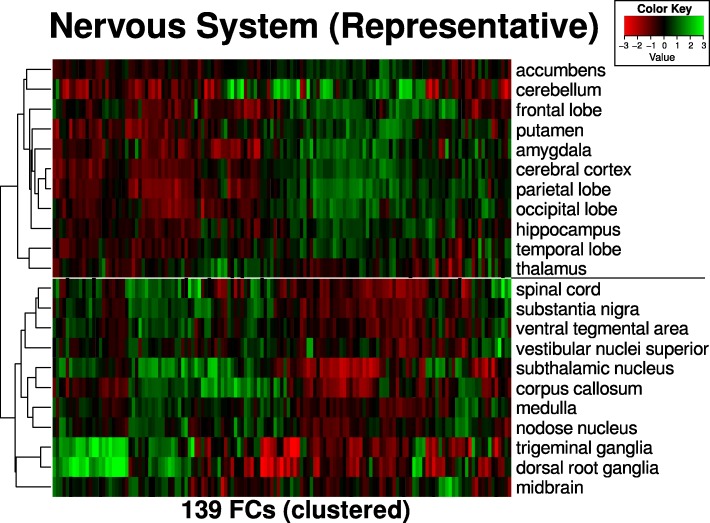
Representative tissue samples from the nervous system (GSE3526). Medoid samples from each tissue type were selected to be the representative for the tissue. The representatives were then clustered using hierarchical clustering with average linkage. The full range of samples can be found in Additional file 1: Figure S3 (no clustering performed, but the order of the tissue types is the same as here). The order of the FCs in the plot can be found in Additional file 1: Text S1

About a third of the samples from GSE3526 were from 22 tissue types belonging to the nervous system, and we performed clustering of the representatives from these tissues (Fig. 6). The clustering displayed underlying anatomical and physiological similarities between the tissues. For instance, the tissues from the three lobes (parietal, occipital and temporal) were grouped together with the cerebral cortex in one major cluster, whereas the other cluster was enriched for tissues from the peripheral nervous system, such as ganglia tissues (trigeminal, dorsal root) and the spinal cord, and most members of the basal ganglia (substantia nigra, subthalamic nucleus, ventral tegmental area).

**Fig. 7:**
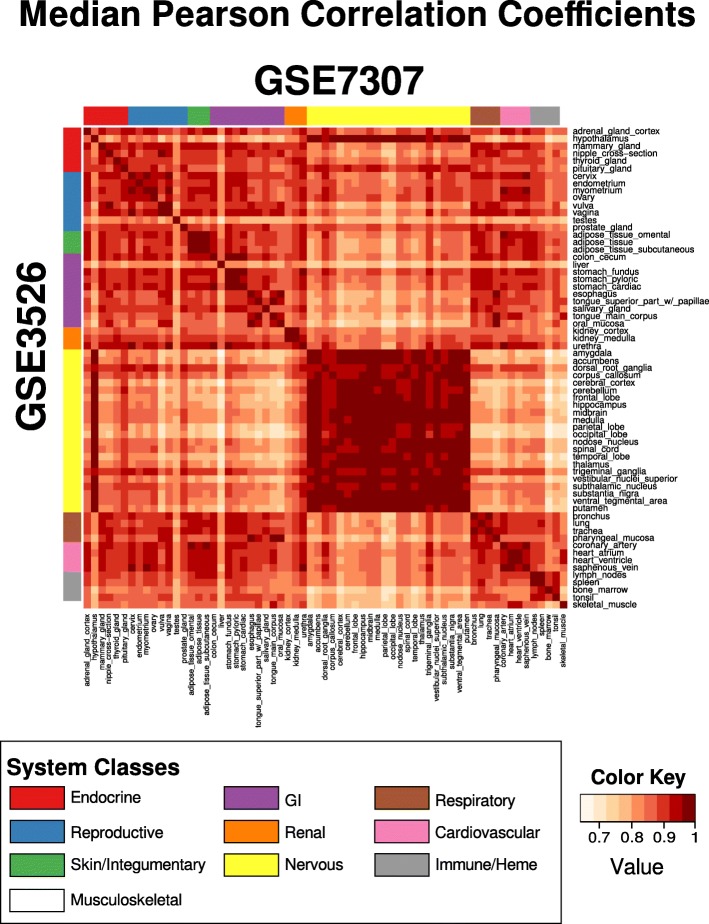
Tissue sample correlations between GSE3526 and GSE7307. Pairwise Pearson correlation coefficients were calculated (in FC space) between samples from GSE3526 and GSE7307. The figure shows the median correlation scores for each GSE/tissue-GSE/tissue pair. High correlation is observed between anatomically-related tissues

There are a total of 65 tissues types that were common to both GSE3526 and GSE7307 based on the annotations in GEO (the tissue types in former is a proper subset of the latter). The median Pearson correlation coefficients (MPC) between tissues from GSE3526 and GSE7307 are shown in Fig. 7. Tissues from the same classes (diagonal of Fig. 7) were highly correlated, with an average MPC of 0.985 and an interquartile range of 0.981 to 0.990. The mean standard deviation across the whole MPC matrix was 0.0269, with an interquartile range of 0.0157 to 0.0346.

### FCs as features for machine learning algorithms

To demonstrate the applicability of our FCs as features for use in machine learning algorithms, we apply our FCs to two different studies (rheumatoid arthritis and leukemia). Additionally, we performed subsampling of the leukemia study to compare how model performances in FC space and full gene space are affected in low-sample settings.

#### GSE71370 (rheumatoid arthritis)

GSE71370 contains three sample types: peripheral blood from rheumatoid arthritis (RA) patients (RAPBM), peripheral blood from healthy patients (HCPBM), and synovial fluid from RA patients (RASFM). Using the standard Affymetrix chip definition file (CDF), we found 6636 DE genes between RASFM and HCPBM, and zero DE genes between RAPMB and HCPMB.

**Fig. 8:**
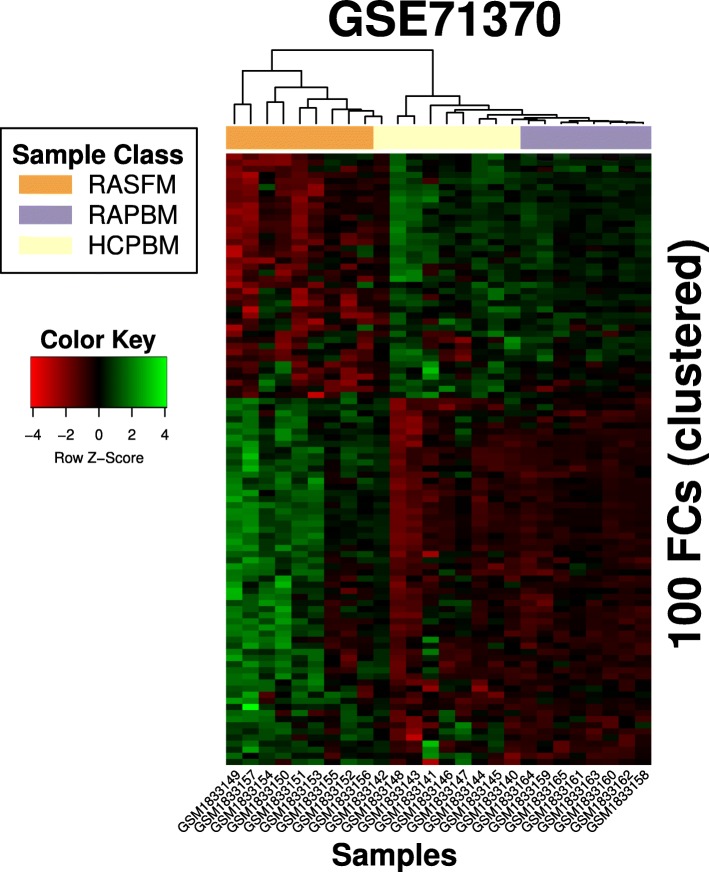
Clustering of GSE71370 samples using 100 FCs. 100 FCs were identified to be DE between the pairwise classes, and were used to perform clustering on the samples. The three sample classes were separated very well by hierarchical clustering (average linkage), with only GSM1833142 appearing to be clustered incorrectly as RASFM. The order of the FCs in the heatmap can be found in Additional file 1: Text S1

There are zero differentially expressed (DE) FCs between RAPBM and HCPBM, 72 DE FCs between RAPBM and RASFM, and 89 DE FCs between RASFM and HCPBM. 61 FCs were common in the latter two sets, resulting in a combined signature of 100 FCs for clustering. Figure 8 shows the clustering results using the signature. A distinct separation between the classes is observed, and the two subgroups from the same tissue type (peripheral blood) are clustered together. There are eleven DE FCs that are unique to the comparison between RASFM and RAPBM. Additional file 1: Figure S4 shows the corresponding clustering results in gene space, and Additional file 1: Table S3 lists the FCs and the corresponding GO annotations. In total, there were 75 unique GO terms that were associated with the selected FCs.

#### GSE13159 (leukemia)

**Fig. 9:**
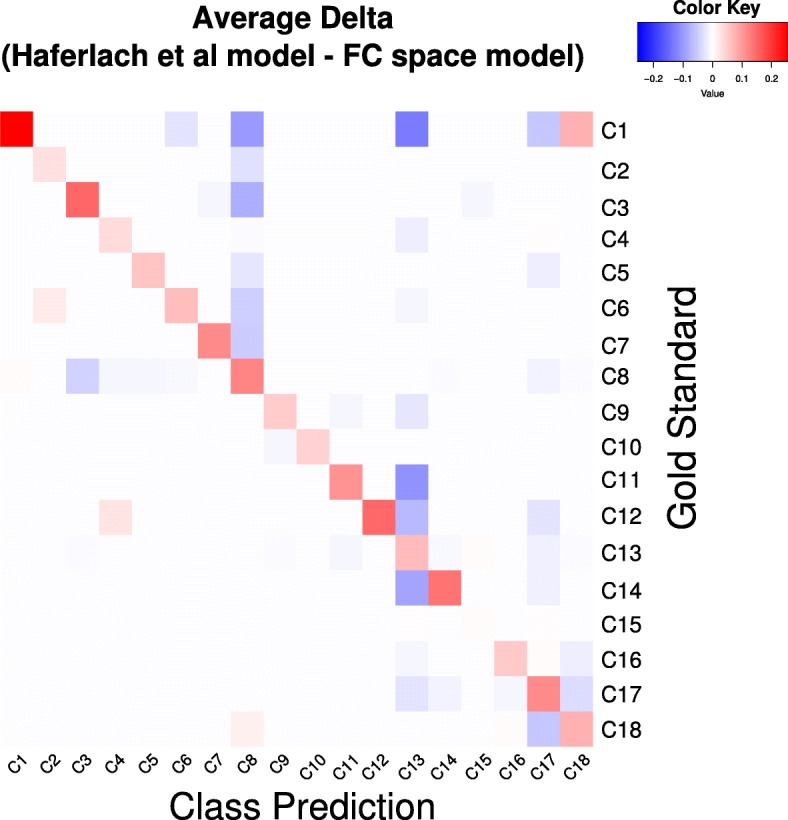
Differences between classification models (GSE12159). The average classification rate from both confusion matrices were normalized row-wise by the total number of samples in each gold standard class. The difference between the matrix from Haferlach et al. and our FC-based model was then computed and represented as a heatmap. Red squares represents higher values in the Haferlach et al. model; in off-diagonals squares, this indicates a higher misclassification by the Haferlach et al. model

GSE13159 contains patient samples from 18 different classes of leukemia. Table 2 shows the confusion matrix of the SVM classification model using data that was projected into our FC space, and Fig. 9 summarizes the average differences between our confusion matrix and that from the original paper (Table 2 in Haferlach et al. [38]) after normalizing for class size. The call rates (CR) achieved by both models are very similar, although the class-wise sensitivity of the model from Haferlach et al. was generally slightly better, averaging at 0.0692 higher than the ones from our model. For half of the 18 classes, the differences between the sensitivities from the two models were insignificant (the median difference is 0.0575), and for class C15, our FC-based model outperformed Haferlach’s model marginally. The misclassification patterns (off-diagonals of the confusion matrices) were similar between both models, although our FC-model misclassified samples as C8 or C13 more frequently.

**Table 2:** FC-based SVM Classification Confusion Matric (GSE13159)

Class Prediction																			Average # of IDC (ties in majority vote)	Total # of Specimens	CR	Sensitivity for Called Specimens
GS/ Call	C1	C2	C3	C4	C5	C6	C7	C8	C9	C10	C11	C12	C13	C14	C15	C16	C17	C18				
C1	6.0	–	–	–	–	0.3	–	2.0	–	–	–	–	2.0	–	–	–	1.0	–	1.7	13	0.872	0.530
C2	–	68.0	–	–	–	–	–	2.0	–	–	–	–	–	–	–	–	–	–	–	70	1.000	0.971
C3	–	–	96.0	–	–	–	1.3	17.0	–	–	–	–	–	–	1.0	–	–	–	6.7	122	0.945	0.833
C4	–	–	–	160.3	–	–	–	2.3	–	–	–	–	7.0	–	–	–	–	1.0	3.3	174	0.981	0.939
C5	–	–	–	–	50.3	–	–	5.7	–	–	–	–	–	–	–	–	1.0	–	1.0	58	0.983	0.883
C6	–	1.3	–	–	–	30.7	–	2.7	–	–	–	–	0.3	–	–	–	–	–	1.0	36	0.972	0.876
C7	–	–	–	–	–	–	25.7	11.7	–	–	–	–	–	–	–	–	–	–	2.7	40	0.933	0.687
C8	–	3.3	16.0	3.3	8.3	1.3	11.0	177.0	–	–	–	–	0.7	1.7	1.0	0.3	4.3	1.0	7.7	237	0.968	0.772
C9	–	–	–	–	–	–	–	–	38.0	–	0.3	–	1.0	–	–	–	–	–	0.7	40	0.983	0.966
C10	–	–	–	–	–	–	–	–	0.3	33.3	–	–	1.0	–	–	–	1.0	–	1.3	37	0.964	0.935
C11	–	–	–	–	–	–	–	–	–	–	25.0	–	3.0	–	–	–	–	–	–	28	1.000	0.893
C12	–	–	–	–	–	–	–	–	–	–	–	26.3	7.7	–	–	–	1.0	–	3.0	38	0.921	0.753
C13	1.0	–	1.7	4.3	–	–	–	3.0	1.0	1.0	3.0	2.7	287.3	12.0	0.3	1.0	20.0	1.0	11.7	351	0.967	0.847
C14	–	–	–	–	–	–	–	–	–	–	–	–	13.7	29.0	–	–	3.3	–	2.0	48	0.958	0.630
C15	0.3	–	1.0	–	–	–	–	–	–	–	–	0.3	0.3	–	444.3	–	–	–	1.7	448	0.996	0.996
C16	–	–	1.0	–	–	–	–	–	–	–	–	–	0.7	–	–	68.0	–	4.0	2.3	76	0.969	0.923
C17	–	–	–	–	–	–	–	–	–	–	–	–	16.7	2.3	–	1.7	160.3	15.0	10.0	206	0.951	0.818
C18	–	–	–	–	–	–	–	–	–	–	–	–	–	–	1.0	0.7	18.0	51.3	3.0	74	0.959	0.723

*Abbreviations*, *IDC* indeterminable calls, *Mean CR* average call rate across the three runs. Diagonal values are the average number of correct prediction results, while off-diagonals are the average misclassifications. Null values are represented by “-” to enhance visual clarity. This confusion matrix is presented in the same format as Table 2 of [38]

**Fig. 10:**
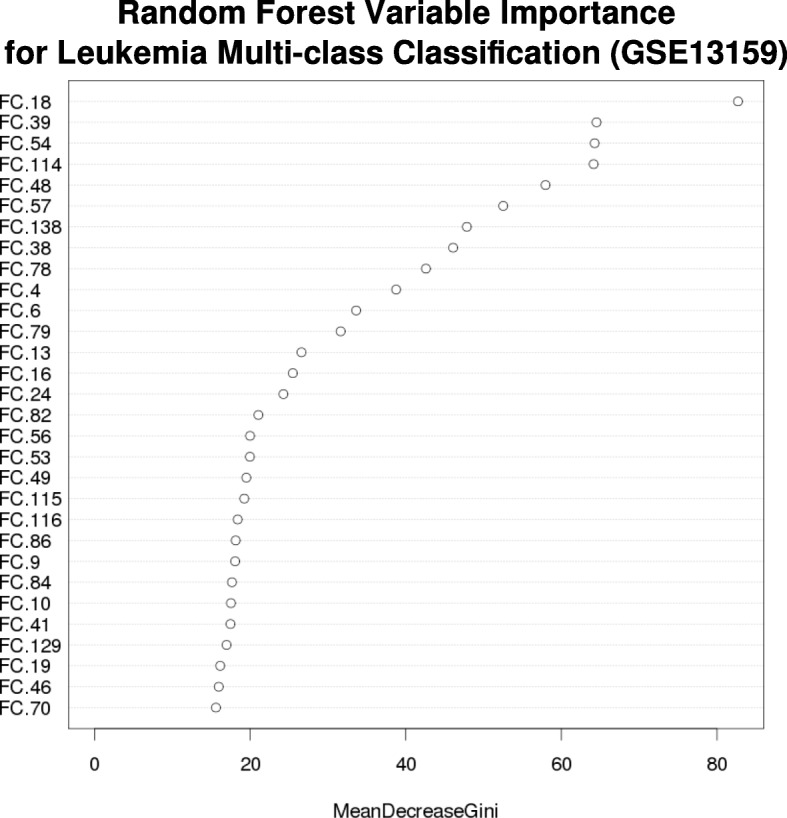
FC importance in leukemia classification, as ranked from a random forest model. A random forest was built using the data from GSE13159, with features being the respective FC scores. FC 18, 39 and 54 were the three most important variables identified by the random forest

*The random forest we built indicated that FC 18, 39 and 54 are the three most important variables* (Fig. 10). *The corresponding GO annotations for the three FCs* (Table 3) *are all related to immune response.*

**Table 3:** GO annotations for DE FCs (GSE13159)

FC	BH-Corrected *p*-value	GO ID	Description
18	0.0102	GO:0050853	B cell receptor signaling pathway
		GO:0002250	adaptive immune response
		GO:0006959	humoral immune response
		GO:0002768	immune response-regulating cell surface ...
		GO:0031295	T cell costimulation
		GO:0030183	B cell differentiation
39	0.0102	GO:0002250	adaptive immune response
		GO:0060394	negative regulation of pathway-restricte...
		GO:0050853	B cell receptor signaling pathway
		GO:0002377	immunoglobulin production
54	0.0109	GO:0014068	positive regulation of phosphatidylinosi...
		GO:0050776	regulation of immune response
		GO:0019371	cyclooxygenase pathway
		GO:0007596	blood coagulation

#### Performance of FC-based models in low sample settings

**Fig. 11:**
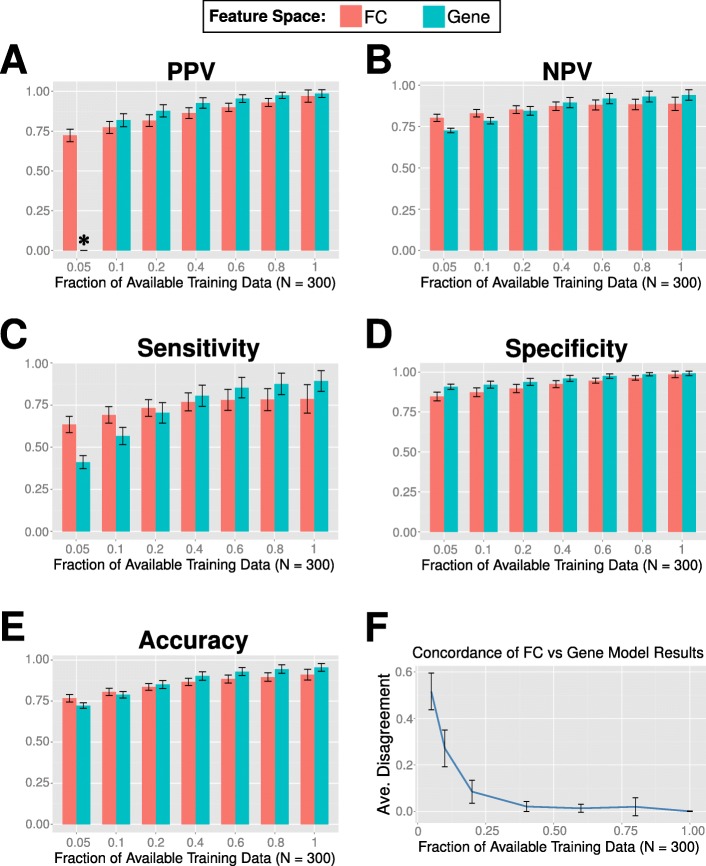
Classification performance at various subsampling ratios. 100 independent simulation runs were performed, each using an independently selected held-out test set. For each run, 200 repeats were performed using different training sets, and we calculated the mean performance metrics across the repeats. At the lowest subsampling percentage (5%), a training set would consist of five C3 and ten C8 samples, both randomly chosen. The performance metrics, averaged over the runs are: (**a**) Positive Predictive Value (i.e. precision), (**b**) Negative Predictive Value, (**c**) Sensitivity (i.e. recall), (**d**) Specificity, (**e**) Accuracy, and (**f**) the amount of agreement between FC and gene based models. Error bars here indicate the standard deviations (across the 100 runs) for the particular metric. *For eleven of the simulation runs (i.e. test sets) at the subsampling percentage of 5%, the gene-space model predicted all negatives in at least one sampling, resulting in an undefined PPV. It should be noted that the FC-based model consistently provided predictions for both classes across all runs; the average PPV for the FC-based model across those eleven runs was 0.714

We subsampled two classes from the leukemia study at various fractions to create datasets of varying sizes. The FC space models had higher NPV, sensitivity and accuracy than the full gene space models when the fraction of training data used was low (Fig. 11b, c and e). Specifically, we observed that the FC-based models had higher sensitivity for subsampling fractions of up to 20% of the full training size (300), and higher accuracy and negative predictive value (*p* < 0.01) for subsampling fractions of up to 10% (both *p* < 0.01). The FC-based model was also able to provide balanced predictions at very low subsample fraction (5%), whereas the gene-based model breaks down and predicts all negatives, resulting in undefined PPV (Fig. 11a) and inflated specificity (Fig. 11d). As the amount of training data used increased, the performance of the models across all evaluation metrics improved, and the predictions of FC- and gene-based models increased in concordance (Fig. 11f). At higher fractions of training data (0.4 and above), the full gene space models dominated in terms of performance.

### FCs retain biological information while regularizing data

We also studied how projection of microarray data into the FC space can regularize data and reduce batch effects in datasets. The MicroArray Quality Control project contains samples from well-controlled, titrated mixtures, allowing for a quantitative assessment of the extent of information loss when projecting into the FC space. The acute myeloid leukemia study, which contains patient sample from three institutes in different geographical locations, provides an avenue to investigate how much batch effects are reduced when projecting into FC space.

#### MicroArray quality control data (MAQC)

**Fig. 12:**
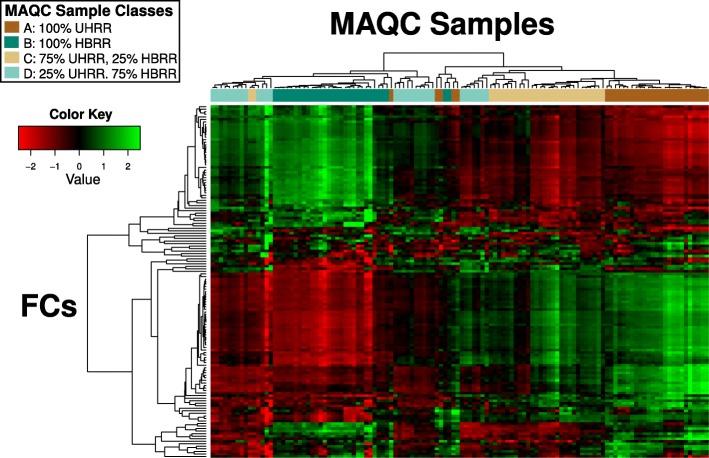
Hierarchical clustering of MAQC samples in FC space. Clustering of MAQC samples (columns) and the FCs (rows) were performed using average linkage. The sample classes were generally preserved across the clusters. The order of both the samples and FCs in the plot can be found in Additional file 1: Text S1

In clustering MAQC samples in our FC space, we observed that the sample classes were generally well preserved (Fig. 12). In particular, if we define the classes based on the compositions of HBRR and UHRR (mega-class 1; A and C, mega-class 2: B and D), and cut the clustering tree to obtain exactly two clusters, the resulting purity of the clusters is 0.9 (the Gini impurity for the clusters are 0.105 and 0.235). The corresponding gene-space tree has a slightly lower purity of 0.875. However, the clustering trees obtained in FC and full gene space were very similar with a cophenetic correlation of 0.863, and certain misclassified samples, such as the set containing A.18, B.6 and B.8, were common to both clustering results (Additional file 1: Figure S2).

#### GSE15434 (acute myeloid leukemia)

**Fig. 13:**
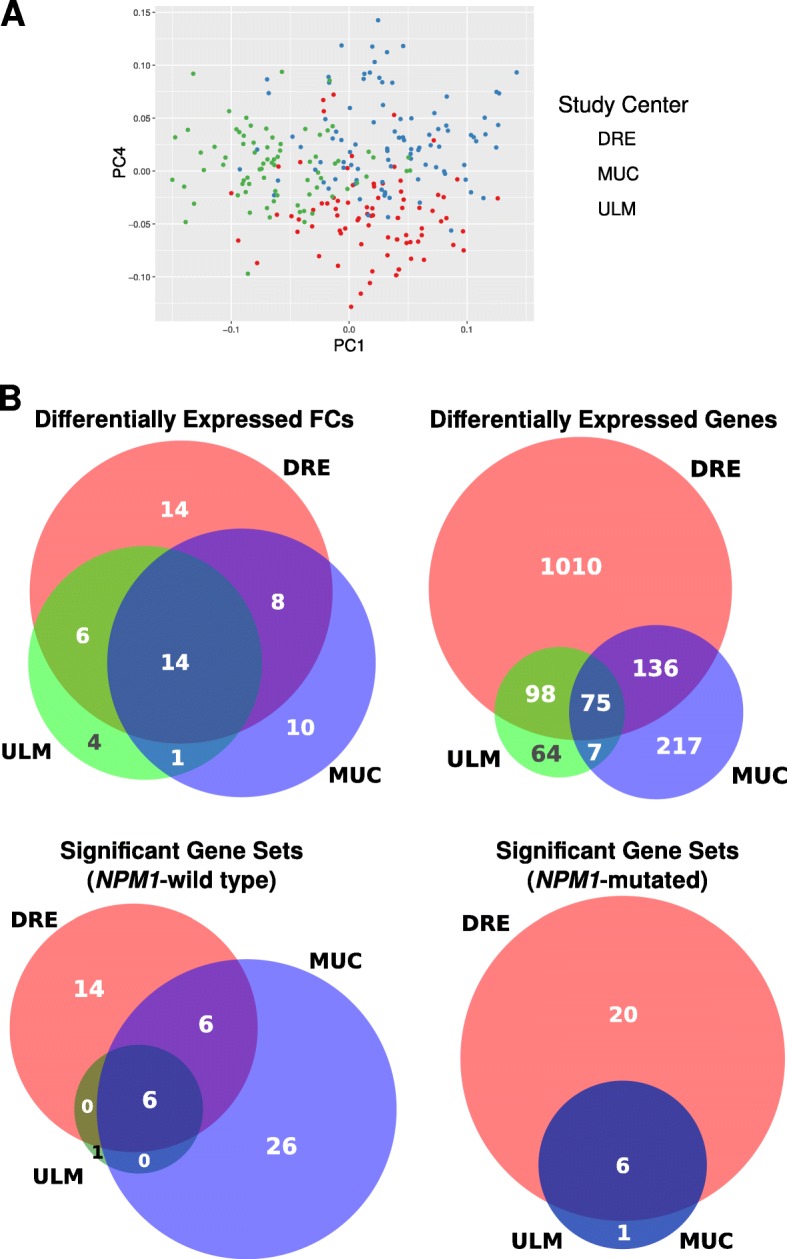
Analysis of batch effects in GSE15434. **a** Gene space data projected on PC 1 and PC 4. **b** Results of differential gene and FC analysis (top), and also GSEA (bottom), on the two phenotypes. GSEA was performed using the C2 (curated gene sets) from MSigDB. The numbers in the Venn diagrams represent the number of genes/gene sets/FCs that were identified for each of those subsets. The Venn diagrams were generated using *BioVenn* [54]

Distinct separation of the AML samples by the three study centers can be observed when the original gene space data is projected on the first and fourth principal components (Fig. 13a). Note that the first few components all separate the study centers to various degrees, and that the choice of using the first and fourth component here was simply to enhance visual clarity.

The patient samples can be grouped into two main phenotypical classes (Table 4). Results of the differential analysis between the two AML phenotype classes (NPM1-mutated and NPM1-wild tpe) were performed in gene and FC space are shown in Fig. 13b (top). The gene sets that were enriched for both classes are also reported in Fig. 13b (bottom).

**Table 4:** Distribution of AML patient samples from the two phenotypic classes across the three test centers in GSE15434

	DRE	MUC	ULM
NPM1-wild type	36	41	36
NPM1-mutated	42	55	41

DRE, MUC and ULM are the three study centers

### Differentially expressed FCs are biologically relevant

We reanalyzed data from two previous studies (rhabdomyosarcoma and dengue virus infection) to demonstrate that DE FCs can provide similar insight to a disease as the conventional DE gene approach.

#### GSE66533 (rhabdomyosarcoma)

**Fig. 14:**
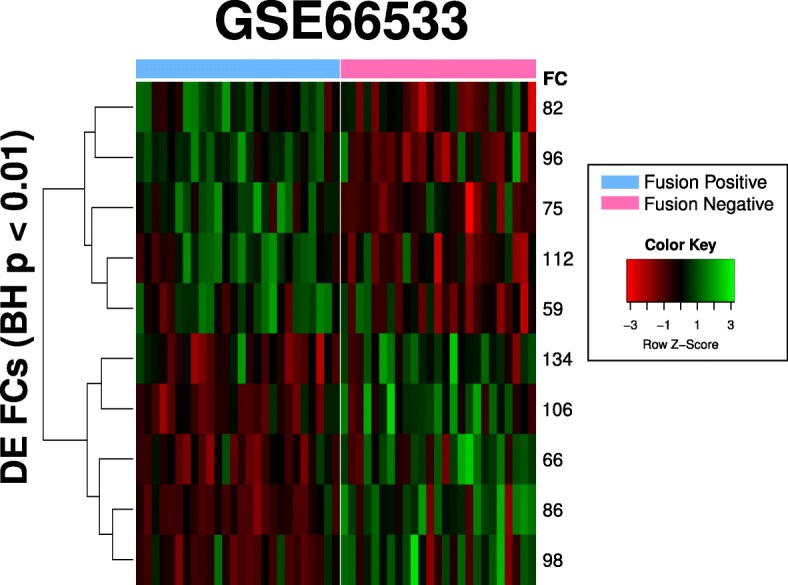
Heatmap of differentially expressed FCs in GSE66533. Ten DE FCs were identified. FC 82, 96 75, 112 and 59 generally had high scores in Fusion Negative samples compared to Fusion Positive samples, while the opposite is true for FCs 134, 106, 66, 86 and 98. The order of the samples in the heatmap can be found in Additional file 1: Text S1

The rhabdomyosarcoma dataset contains patient samples that are either PAX3-FOX01 Fusion-Positive or Fusion-Negative, and DE genes had been previously reported between the two groups. Ten FCs were found to be DE at the 0.01 level (*p*-values were BH-corrected). The Fusion-Positive samples generally had higher FC scores in FCs 59, 75, 82, 96 and 112, and lower scores in 66, 86, 98, 106 and 134 as compared to the Fusion-Negative samples (Fig. 14). The GO terms associated with those FCs are listed in Table 5; FCs 89 and 112 do not have associated GO terms.

**Table 5:** GO terms associated with DE FCs for GSE66533

FC	BH-Corrected *p*-value	GO ID	Description
86	0.000946	GO:0061551	trigeminal ganglion development
		GO:0001706	endoderm formation
		GO:0030574	collagen catabolic process
		GO:0022617	extracellular matrix disassembly
75	0.00108	GO:1990440	positive regulation of transcription fro...
		GO:0006564	L-serine biosynthetic process
		GO:0042149	cellular response to glucose starvation
		GO:0036499	PERK-mediated unfolded protein response
		GO:0070059	intrinsic apoptotic signaling pathway in...
		GO:0002523	leukocyte migration involved in inflamma...
		GO:0030593	neutrophil chemotaxis
		GO:0070488	neutrophil aggregation
96	0.00140	GO:0035860	glial cell-derived neurotrophic factor r...
		GO:1900028	negative regulation of ruffle assembly
106	0.00140	GO:0010903	negative regulation of very-low-density ...
		GO:0043627	response to estrogen
		GO:0030199	collagen fibril organization
		GO:0005975	carbohydrate metabolic process
		GO:0044281	small molecule metabolic process
		GO:0042632	cholesterol homeostasis
66	0.00193	GO:0051965	positive regulation of synapse assembly
		GO:0031290	retinal ganglion cell axon guidance
		GO:0030574	collagen catabolic process
		GO:0022617	extracellular matrix disassembly
		GO:0030198	extracellular matrix organization
112	0.00266	-	-
59	0.00992	-	-
82	0.00992	GO:0035456	response to interferon-beta
		GO:0051607	defense response to virus
		GO:0045669	positive regulation of osteoblast differ...
98	0.00992	GO:0071294	cellular response to zinc ion
		GO:0071276	cellular response to cadmium ion
		GO:0001525	Angiogenesis
		GO:0035025	positive regulation of Rho protein signa...
134	0.00992	GO:2000373	positive regulation of DNA topoisomerase...

We also identified 38 and 43 “neighbors” for the Fusion-Positive and Fusion-Negative group respectively. 32 of these “neighbors” were common to both groups. Note that the maximum correlation between samples from the two classes was 0.993, and the minimum was 0.890. The mean correlation between the two classes was 0.970.

Using our “Human Tissue Compendium”, we found that the highest correlation of the samples were with the tissue types “myometrium” (top hit for 48 samples, mean correlation is 0.865), followed by “endometrium” (top hit for five samples), “deltoid muscle” (top hit for four samples) and “synovial_membrane” (top hit for one sample).

#### E-MTAB-3162 (dengue virus exposure)

**Fig. 15:**
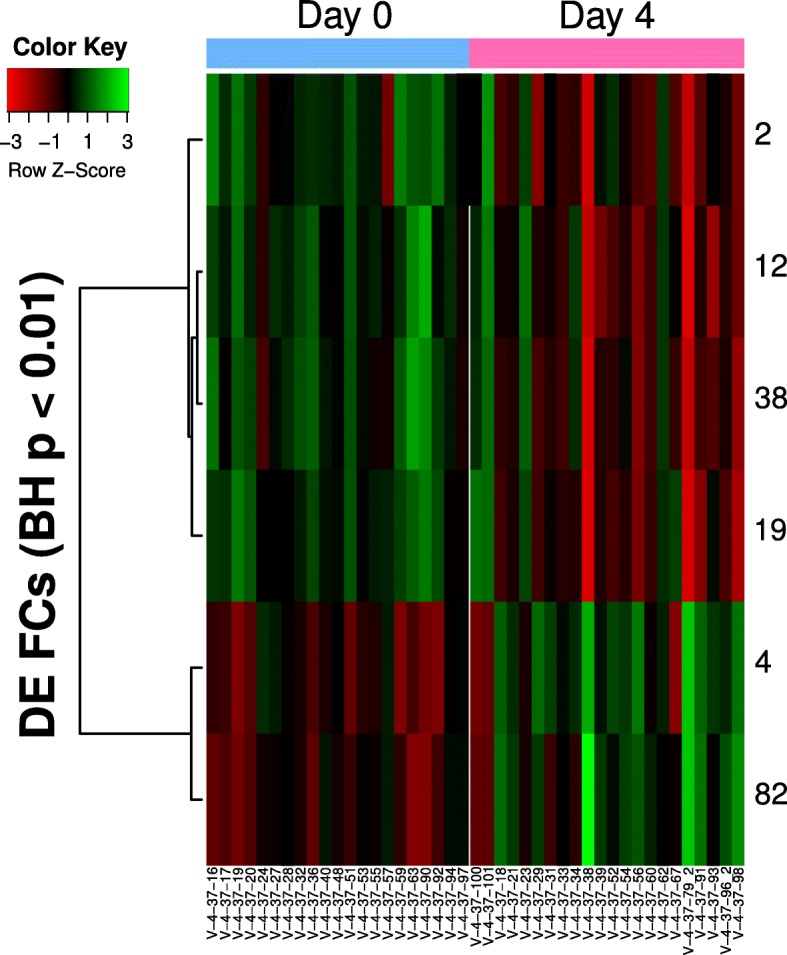
Heatmap of differentially expressed FCs in dengue patients from different exposure times. (E-MTAB-3162). Six DE FCs were identified, with Day 4 patients having lower scores in FC 2, 12, 38 and 19 compared to Day 1 patients. Day 1 patients had lower scores in FCs 4 and 82

The dengue viral study contained Day 0 and Day 4 patients, and the two time points were previously reported to have different gene expression profiles due to the dynamics of viral response. Six FCs were differentially expressed between in Day 0 and Day 4 dengue patients (Fig. 15), which collectively mapped to 150 unique GO terms (Additional file 1: Table S2). Some of the GO terms pertain directly to dengue disease phenotype, such as “platelet activation”, “platelet degranulation” and “blood coagulation”. There is a clear enrichment for immune response GO terms, with “immune response” (GO:0006955) appearing in five of the six FC’s GO annotations; the exception is FC 4, which is instead enriched for cell division process terms. Other GO terms related to immune response, such as “inflamatory response” (GO:0009954), “neutrophil chemotaxis” (GO:0030593) and “T cell receptor signaling pathway” (GO:0050852), also appear in the annotations for at least two different FCs. The GO slim terms in Table 6 provides a summarized view of the biological processes covered by each FC, and shows the subtle differences between them. For instance, FC 12 is the only immune-related FC amongst the five that also focuses on cell adhesion and proliferation, whereas FC38 is almost completely dedicated to only the immune system process. FC2 has nine GO annotations that map to the GO slim term for symbiosis/parasitism, and is also the only DE FC that has GO terms specifically related to viral responses.

**Table 6:** GO Slim terms for DE FCs in E-MTAB-3162

FC	BH-Corrected *p*-value	GO Slim ID	Number of Children ID	Description
2	0.00933	GO:0002376	9	Immune system process
		GO:0044403	5	Symbiosis, encompassing mutualism through parasitism
		GO:0008150	5	Biological process
		GO:0006950	4	Response to stress
		GO:0007165	3	Signal Transduction
4	0.00933	GO:0006259	18	DNA metabolic process
		GO:0051276	12	Chromosome organization
		GO:0009058	11	Biosynthetic process
		GO:0006950	10	Response to stress
		GO:0007049	9	Cell cycle
		GO:0140014	8	Mitotic nuclear division
		GO:0007059	7	Chromosome segregation
		GO:0007010	7	Cytoskeleton organization
		GO:0000278	7	Mitotic cell division
		GO:0000003	3	reproduction
		GO:0006464	2	cellular protein modification process
		GO:0042592	2	Homeostatic process
		GO:0051301	2	Cell division
12	0.00933	GO:0002376	16	Immune system process
		GO:0006950	8	Response to stress
		GO:0007165	8	Signal Transduction
		GO:0008150	6	Biological process
		GO:0016192	4	vesicle-mediated transport
		GO:0007155	3	Cell adhesion
		GO:0008283	2	Cell proliferation
19	0.00933	GO:0002376	11	Immune system process
		GO:0006950	11	Response to stress
		GO:0007165	6	Signal Transduction
		GO:0008150	5	Biological process
		GO:0006810	3	Transport
		GO:0009058	2	Biosynthetic process
38	0.00933	GO:0002376	4	Immune system process
82	0.00933	GO:0008150	2	Biological process

The full list of GO annotations for the DE FCs can be found in Additional file 1: Table S2. The table here presents all mapped GO slim terms that have at least one child annotation in the full list for each FC

## Discussion

The use of gene set enrichment analysis (GSEA) is ubiquitous today in transcriptomic analysis, and the growing number of gene sets (signatures) in the MSigDB repository enables a better characterization of biological processes. There is inherent subjectivity in GSEA, however, due to the users’ choice of signatures. Liberzon et al. [6] suggest that a more consistent and reliable approach to GSEA would be to default to the fifty signatures they identified and collected in the H collection of MSigDB. These signatures were based on a careful evaluation of all collections in MSigDB, and were meant to encompass key biologically relevant gene sets. In contrast to such a top-down construction, we propose leveraging the large amount of microarray data accumulated over the past two decades to employ data-driven methods in identifying transcriptomic modules. While the full compendium contains 97,049 arrays, we ultimately identified 2726 representative arrays, from which we derived 139 transcriptomic modules (denoted as functional components, FCs). It does not escape our notice that the drastic reduction in dimensionality is analogous to the reduction of the 17,779 signatures in MSigDB to the 50 signatures advocated by Liberzon et al. This also concurs with current literature [10–12] that despite the large size of the human transcriptome, most biological phenotypes can be fully captured in far fewer dimensions. Preliminary work by our group [19] proposed a set of 423 FCs based on the available data in GEO as of May 2008, and applied it to an AML study involving parthenolide treatment (GSE7538). Despite having far fewer components in our current FC space, we were also able to capture the biological variance and found a set of 19 DE FCs (Additional file 1: Figure S5) for that study. The FC with the lowest BH-corrected *p*-value (FC 79) was also found to be involved in inflammatory and immune responses.

Although we retained only the leading 139 components of the whitened data, ICA convergence on the tall matrix took close to 2000 iterations to reach the preset threshold. We used multiple repeats and also subsampling of the full compendium to evaluate the results, and found that our FCs were well-converged and reproducible. Many of the FCs also had GO annotations for distinct pathways and biological processes, and a few FCs corresponded directly with some of the gene signatures in MSigDB’s H collection (Fig. 5). We note that FCs without clear GO annotations may indicate yet-undiscovered biological pathways, or a higher level grouping that involved multiple pathways. Analysis of the active genes also indicated that most genes are not promiscuous; most genes are active in less than three different FCs (Fig. 4). The most promiscuous gene was AKR1C3 (Entrez ID 8644), which encoded an enzyme from the aldo/keto reductase superfamily. The gene is primarily responsible for the metabolism of prostaglandin and some sex hormones, and is included in pathways from KEGG and REACTOME such as arachidonic acid metabolism, ovarian steroidogenesis and signaling by retinoic acid. As a gene involved in metabolism, it also affects the dosages of certain drugs such as warfarin [45]. More recently, it has been implicated in some cancer studies [46, 47] due to its role in cell growth/differentiation.

Despite the huge reduction in feature space, our FCs are able to capture critical biological features of a dataset and performed well when used in subsequent classification task. In the rheumatoid arthritis study (GSE71370), Rajasekhar et al. noted in their paper [48] that the rheumatoid arthritis peripheral blood samples (RAPBM) and healthy control samples (HCPBM) were highly similar with no DE genes observed, while there were 3033 DE genes between RASFM and HCPBM. However, we achieved a nearly-perfect (only one healthy sample was misclassified) separation of the three classes in the study using only a simple hierarchical clustering with 100 of our FCs as features (Fig. 8). In contrast, the clustering obtained using the set of 6636 differentially expressed probes between RASFM and HCPBM (at 0.05 FDR) was strictly inferior and produced two additional misclassifications (Additional file 1: Figure S4). Our FCs also performed well as features in more complex machine learning models. In the leukemia study (GSE13159), Haferlach et al. obtained a feature set of 3556 probesets by combining the top 100 DE probesets identified across each of the pairwise tests across the 18 leukemia classes. The feature set was then used to train pairwise SVM models, and samples were classified based on a max-vote scheme using all models. We repeated their procedures, but used our full set of 139 FCs as features in place of their manually defined feature set for the classification task. We did not report specificity as the computation method was unclear from the original paper; additionally, since specificity focuses on the negative classification, it is not informative here when the number of negative classes can be easily inflated depending on one’s interpretation of what qualifies as a negative class. The misclassification patterns (off-diagonal, non-zero elements of Table 2) generally follow the same trends as what the authors reported with slight differences in the class predictions for C8 and C13 (Fig. 9). While our classification sensitivity were generally lower than the reported numbers in the original paper, our choice of features were agnostic to the data values and were not primed to maximize the difference between the classes as the authors did with their pairwise feature selection algorithm. Additionally, the largest deviation between the reported sensitivities occurs when the sample size is small, such as that of C1 (thirteen samples). For classes with larger sample sizes, the difference between the sensitivities reported by our FC-based model and the original paper are less significant. For instance, the sensitivities reported for the two largest classes, C13 and C15, in the original paper are 0.890 and 0.998 respectively. We report corresponding sensitivities of 0.850 and 1.00, the latter of which is higher than the original paper’s. We posit that in the cross-validation procedure, classes with small sample sizes may not be included in the training set within certain folds, resulting in poor test set prediction results.

An additional benefit of using the FC scores as features is that it is easy to train tree-based models that are more interpretable than SVM. Using the same leukemia dataset, we trained a random forest, which would have been challenging to do if the full gene space (20,089 genes as features) was used instead. We identified FC18, FC 39 and FC54 as the top three most important features (Fig. 10) in separating the various leukemia classes, based on the mean decrease in Gini index. Although the three FC’s share a number of GO annotations and are all related to inflammation (Table 3), we note that when analyzed collectively, the described processes are subtly different; the GO annotations for FC18, FC39 and FC 54 suggest the biological processes of B-cell maturation, B-cell production and small molecule immune modulators respectively. In particular, genes CD22, CD19, CD79A and HLA-DRA (Entrez IDs 4861, 5929, 5135 and 7978 respectively), which Haferlach et al. identified as members of a “virtual immunophenotype” (Fig. 3 of [38]) for leukemia classification, are all active genes in the most important FC (FC18).

FC-based models are also robust in low-sample count settings. By subsampling two related leukemia classes from GSE13159 at various percentage levels, we obtained estimates of how well a classification model would perform if trained only on limited data. We note that the FC-space models generally perform better than the corresponding full gene-space models when the subsampling percentage is low, displaying higher NPV, sensitivity and accuracy (Fig. 11). Although the PPV for the gene-based model is higher at the subsampling percentage of 5%, the gene-based model did not provide and positive class prediction in a number of simulations, leading to undefined PPV for more than a tenth of the 100 simulation runs. In contrast, the FC model performance was consistent throughout the simulations and was not affected by the specific choice of test set in particular runs. Since the gene-based model predicted all negatives in those runs, this correspondingly boosted its specificity and gave an inflated view of its actual performance. This is also evidenced by the low concordance of the models at low subsampling percentages; at 5%, the FC-based models produced outputs that were statistically different from their gene-based model counterparts in more than half of the simulations. Performance metrics for the gene-based models are generally more sensitive to the subsampling percentage, and display huge gains at each size increment as opposed to the modest ones from the FC-based models. At the maximum subsampling percentage (100%), gene-based models consistently outperformed their FC-based counterparts, highlighting the tradeoff between robustness in low sample count settings and better fit at high sample count settings. We note, however, that most studies in GEO have sample sizes of that are less than fifty samples (corresponding to a subsampling percentage of less than 17%). The maximum subsampling percentage in our study corresponds to a training sample size of 300 samples, a luxury that is often not available in the majority of typical clinical studies where cost and tissue availability are significant limitations. As of June 2017, there are 4627 GSEs in GEO from the HG-U133 Plus 2.0 platform (GPL570), of which 686 have less than five samples each, 1146 have between five to ten samples and 1981 have ten to 50 samples. In such “small data” studies, we advocate the use of our FCs as a way to utilize the information in the data efficiently.

The use of FCs can also help in denoising the transcriptomic data by emphasizing changes in the active genes. Clustering of MAQC data in both gene and FC space were observed to be highly correlated, although the latter displayed slightly higher cluster purity. We also considered a set of samples from AML patients collected across three different study centers in Germany (GSE15434) to study how the batch effects (Fig. 13a) are affected by FC projection. The patient samples were organized into two phenotypes: those with NPM1 mutations and those with the wildtype NPM1 [49]. A typical differential gene expression analysis reveals that the DE genes identified from the three centers have a small overlap relative to the number of DE genes that were unique to each study center (Fig. 13b). In particular, the number of DE genes unique to the Dresden center was twelve folds larger than the number in the overlap. Similarly, significant gene sets associated with the two phenotypes using GSEA showed poor inter-center parsimony. The DE FCs, however, showed greater concordance between the three centers.

The FCs we have found are also biologically relevant, and the traditional workflow for identifying DE genes can be done analogously to identify DE FCs. In GSE71370, we compared rheumatoid arthritis synovial fluid samples (RASFM) indirectly with HCPBM (healthy control synovial fluid samples were not included in the original study) and identified eleven DE FCs, which were associated with 75 unique GO annotations. Many of them were immediately recognizable as markers of inflammation and apoptosis. A few also had GO annotations that corresponded to lipopolysaccharides (e.g. GO:0071222), which have been reported [50] to be an inducer of microRNA miR-155. This corroborates the role of miR-155 in the disease progression: overexpression of miR-155 is associated with reduced production of matrix metalloproteinases and is believed to therefore reduce inflammation [51], but it also prolongs the presence of CD14+ in inflamed tissues which can aggravate rheumatoid arthritis [48]. In the rhabdomyosarcoma study (GSE66533) where 1002 DE genes were originally identified between the two groups (PAX3-FOX01 Fusion-Positive and Fusion-Negative), we identified ten DE FCs whose GO annotations (Table 5) included typical cancer-related terms such as angiogenesis, extracellular matrix catabolism and immune response. We also demonstrated the use of our FCs in a viral infection study: E-MTAB-3162 contains patient samples from a dengue study performed by van de Wag et al. [52], with the two sample groups determined by exposure time (day 0 and day 4). Differential profiles between the patient groups (Fig. 4 of [52]) were identified by van de Wag et al. by analyzing the transcriptome via pathways and gene sets found in Reactome. In particular, they found that Day 0 patients had expression levels that were enriched for cytokine signaling, innate immune system, interferon signaling and the complement immune system, as compared to the healthy controls. Day 4 patients, when compared to the Day 0 patients, were instead enriched for adaptive immune system responses, cell cycle, DNA repair and metabolism. Here, we identified six DE FCs between the Day 0 and Day 4 patients, and found that GO annotations for these FCs were aligned with the reported trends. For instance, FC 2, 12 38 and 19, which had higher FC scores in Day 0 patients compared to Day 4 patients (Fig. 15), contained GO annotations (Additional file 1: Table S2) that were enriched for immune response and interferon pathways. In particular, “complement activation” is a GO annotation that was found in FC 12 and 38, “adaptive immune response” was found in FC 12 and 19, and “innate immune response” was found in FC 2, 12 and 19. Notably, FC 4, which had higher scores in Day 4 patients as compared to Day 0 patients, was associated with gene expression and cell cycle. It had also been previously reported [53] that genes CXCL10 (or IP-10, Entrez ID: 3627) and CXCL11 (or I-TAC, Entrez ID: 6373), both of which are members of the NF-kB pathway, are highly upregulated upon dengue infection. We note that in the six identified DE FCs, CXCL10 is an active gene in three of them (FC2, FC38 and FC82), and CXCL11 is an active gene in two of them (FC2 and FC38). This is significant when one considers that both CXCL10 and CXCL11 are only active in eight FCs each (out of the possible 139 FCs), and co-occur as active genes in only five FCs.

The compact representation of the full gene values enables a quick scan across GEO datasets to search for similar samples (“neighbors”) based on gene expressions. We demonstrate this search process using the samples in GSE66533, obtaining 43 “neighbors” for the Fusion-Negative group and 38 “neighbors” for the Fusion-Positive group. We note, however, that the rhabdomyosarcoma samples are highly correlated between the two groups (mean Pearson correlation: 0.970). This means that the “nearest” sample to a member of the Fusion-Positive group is also likely to be “near” members of the Fusion-Negative group. Of the “neighbors” we obtained, 30 were common to both groups. Although leiomyosarcome samples seem to be exclusively found in Table 7 and synovial sarcoma in Table 8, this division is superficial as they were prevalent as “neighbors” common to both groups. Using the Human Tissue Compendium, we also found that most of the samples were similar to connective tissues such as myometrium, endometrium and deltoid muscle, suggesting that despite the diseased nature of the rhabdomyosarcoma samples, the biological signals from the originating tissues remained strong.

**Table 7:** Neighbors unique to Fusion-Positive Samples

GSM	Freq	MinCor	GSM description
GSM411049	17	0.939998	Leiomyosarcome (trunk wall)
GSM525975	16	0.935578	Liposarcoma – dedifferentiated
GSM525840	15	0.938809	Liposarcoma – dedifferentiated
GSM525978	14	0.943752	Leiomyosarcome (trunk wall)
GSM411126	14	0.937972	Liposarcoma – dedifferentiated
GSM525837	13	0.943554	Unlisted sarcoma in trunk wall

Abbreviations: Freq, frequency of samples which had this neighbor; MinCor, minimum correlation between those samples and the neighbor

**Table 8:** Neighbors unique to Fusion-Negative Samples

GSM	Freq	MinCor	GSM description
GSM506629	19	0.943743	Monophasic synovial sarcoma
GSM506648	17	0.944286	Monophasic synovial sarcoma
GSM855589	15	0.946213	Ewing-negative poorly differentiated small round cell sarcoma
GSM506642	14	0.95061	Biphasic synovial sarcoma
GSM855590	14	0.944746	Ewing-negative fusiform cell sarcoma
GSM506646	13	0.950141	Monophasic synovial sarcoma
GSM506655	13	0.948231	Monophasic synovial sarcoma
GSM506653	13	0.945084	Poorly differentiated synovial sarcoma
GSM1305464	12	0.947481	Mammary gland tumor cell
GSM526066	12	0.946055	Undifferentiated sarcoma in extremities
GSM506632	12	0.945403	Monophasic synovial sarcoma

*Abbreviations Freq* frequency of samples which had this neighbor, *MinCor* minimum correlation between those samples and the neighbor

## Conclusions

Our FC methodology negates the need for selection of genes to be used in classifier models, allowing it to be easily implemented in smaller studies where data is scarce. Its superior performance in classification tasks at sample sizes common to clinical studies (less than 50 samples) suggest that it is beneficial to perform analysis in FC space. We have written up an *R* package (*humanFC*) that contains the FC loadings and other functions for users to perform simple analysis. The package can be downloaded at https://simtk.org/projects/humanfc

## Additional file

Additional file 1: **Text S1.** Axis labels for Figures in Paper. **Table S1.** MeSH Annotations of Representative Compendium Samples. **Figure S1.** KNN-kneeplots from Full Compendium. **Figure S2.** Tanglegram of FC and Gene space dendrograms (MAQC data). **Figure S3.** Heatmap of all samples in nervous system (GSE3536). **Figure S4.** Clustering of GSE71370 samples using 6636 DE genes. **Figure S5.** Reanalysis of GSE7538 (Parthenolide study used by Engreitz et al.). **Figure S6.** Number of unique GO terms versus number of leading principal components from PCA. **Table S2.** Full GO annotations for DE FCs in E-MTAB-3162. **Table S3.** Full GO annotations for DE FCs in GSE71370. (ZIP 1335 kb)

## Abbreviations

AML: Acute myeloid leukemia
FC: Functional Components
GEO: Gene Expression Omnibus
GSE: GEO series record
GSEA: Gene set enrichment analysis
GSM: GEO sample record
ICA: Independent component analysis
