# Supplementary material for: Data-driven human transcriptomic modules determined by independent component analysis
Source: BMC Bioinformatics. 2018 Sep 17;19:327. doi: 10.1186/s12859-018-2338-4 (PMC6142401; doi:10.1186/s12859-018-2338-4)
Supplement: Supplementary file 1 — Text S1. Axis labels for Figures in Paper. Table S1. MeSH Annotations of Representative Compendium Samples. Figure S1. KNN-kneeplots from Full Compendium. Figure S2. Tanglegram of FC and Gene space dendrograms (MAQC data). Figure S3. Heatmap of all samples in nervous system (GSE3536). Figure S4. Clustering of GSE71370 samples using 6636 DE genes. Figure S5. Reanalysis of GSE7538 (Parthenolide study used by Engreitz et al.). Figure S6. Number of unique GO terms versus number of leading principal components from PCA. Table S2. Full GO annotations for DE FCs in E-MTAB-3162. Table S3. Full GO annotations for DE FCs in GSE71370. (ZIP 1335 kb) [file 12859_2018_2338_MOESM1_ESM.zip › SupplementaryInformation.pdf]

Supplementary Information:

Data-driven Human Transcriptomic Modules Determined by Independent Component Analysis

Weizhuang Zhou[1] and Russ B. Altman[1,2,*]

[1]Department of Bioengineering, [2]Department of Genetics, Stanford University, Stanford, CA 94305 USA.

*To whom correspondence should be addressed. (rbaltman@stanford.edu)

Table of Contents

Note on FCs as loading vectors 3

Text S1: Axis labels for Figures in Paper 4

Table S1: MeSH Annotations of Representative Compendium Samples 6

Figure S1: KNN-kneeplots from Full Compendium 9

Figure S2: Tanglegram of FC and Gene space dendrograms (MAQC data) 10

Figure S3: Heatmap of all samples in nervous system (GSE3536) 11

Figure S4: Clustering of GSE71370 samples using 6636 DE genes 12

Figure S5: Reanalysis of GSE7538 (Parthenolide study used by Engreitz et al) 13

Figure S6: Number of unique GO terms versus number of leading principal components from PCA 14

Table S2: Full GO annotations for DE FCs in E-MTAB-3162 15

Table S3: Full GO annotations for DE FCs in GSE71370 19

Note on FCs as loading vectors

Projection of data into the FC space is analogous to PCA; the loading vectors here are the FCs, and the FC scores are the projected values. It should be noted that although the loadings for each FC have mean zero and unit standard deviation, the Euclidean norm of each loading vector is not unitary. This has no implications on differential expression (DE) analysis or statistical tests such as the t-test where the FC scores would be normalized. The scale of FC scores also has no implications for tree based classification methods such as random forests. However, the scale becomes significant when the FC scores are treated collectively as a vector in FC-space. This is particular obvious in clustering when the Euclidean norm is used to evaluate distances between projected samples, since FCs with larger scores will distort the distance. Additionally, some implementations of machine learning methods assume that the input has already been pre-normalized. In these cases, a normalized version of the FCs is necessary and we have included it in our R package. We use this normalized version explicitly in the analysis of GSE13159 and GSE15434, but default to the original FC loadings for all other cases.

Text S1: Axis labels for Figures in Paper

For visual clarity, labels were removed if there were too many rows/columns. The following provides the exact labels in the order they appear in the paper figures.

Figure 5

Column labels (samples*, left to right):

D.2, D.1, D.4, D.11, D.14, D.22, D.23, D.25, D.24, C.11, C.13, D.12, D.20, D.19, D.16, B.25, B.24, B.21, B.22, B.23, B.13, B.15, B.17, B.18, B.12, B.14, B.11, B.20, B.26, B.28, B.29, B.30, B.1, B.4, B.5, B.2, B.3, B.19, B.16, B.27, B.7, B.10, B.9, A.16, D.18, D.21, D.13, D.5, D.3, D.28, D.26, D.29, D.30, D.27, A.21, A.18, B.8, B.6, A.17, A.19, D.8, D.9, D.10, D.6, D.7, D.17, D.15, C.22, C.14, C.12, C.1, C.4, C.3, C.2, C.23, C.25, C.15, C.24, C.5, C.6, C.7, C.9, C.10, C.8, C.18, C.17, C.19, C.20, C.21, C.26, C.28, C.30, C.27, C.29, C.16, A.14, A.11, A.1, A.2, A.4, A.3, A.5, A.15, A.12, A.13, A.22, A.23, A.25, A.24, A.9, A.8, A.6, A.7, A.10, A.20, A.27, A.30, A.26, A.29, A.28

Row labels (FC, bottom to top)

130, 93, 89, 90, 36, 128, 101, 136, 100, 120, 44, 99, 98, 92, 69, 74, 122, 62, 53, 52, 64, 95, 124, 84, 139, 9, 125, 132, 42, 68, 78, 106, 87, 2, 31, 71, 49, 22, 5, 96, 79, 30, 105, 13, 65, 50, 15, 70, 47, 56, 3, 48, 135, 4, 73, 61, 129, 115, 138, 126, 54, 83, 29, 116, 46, 81, 121, 107, 75, 117, 77, 113, 45, 23, 86, 17, 10, 137, 40, 80, 20, 102, 94, 51, 6, 123, 35, 18, 57, 88, 82, 63, 103, 119, 19, 134, 76, 59, 112, 127, 60, 131, 97, 133, 85, 110, 27, 114, 1, 72, 91, 14, 26, 104, 118, 109, 11, 16, 58, 111, 33, 21, 32, 24, 39, 12, 43, 25, 67, 41, 34, 108, 55, 66, 38, 37, 7, 8, 28

*Mapping between actual GSM IDs to sample IDs here can be found in the supplementary file (GSM5350_meta.txt)

Figure 6

Column labels (FC, left to right)

99, 66, 29, 14, 53, 97, 59, 76, 6, 64, 11, 10, 138, 72, 80, 123, 112, 87, 125, 31, 18, 8, 78, 134, 37, 54, 95, 106, 131, 40, 89, 128, 83, 135, 79, 32, 7, 60, 63, 103, 114, 28, 75, 77, 61, 33, 48, 67, 68, 111, 96, 41, 25, 101, 58, 55, 108, 86, 85, 51, 88, 27, 43, 44, 133, 120, 22, 65, 132, 82, 20, 124, 119, 34, 110, 115, 118, 69, 98, 73, 127, 93, 90, 130, 36, 107, 26, 15, 30, 13, 35, 39, 84, 71, 52, 1, 109, 3, 121, 24, 57, 17, 38, 46, 139, 81, 23, 12, 19, 92, 117, 74, 100, 9, 129, 4, 94, 50, 70, 113, 116, 42, 126, 56, 122, 91, 2, 105, 16, 104, 47, 5, 21, 136, 49, 45, 137, 62, 102

Figure 8

Column labels (Samples, left to right)

A3-11, A3-14, A3-4, A3-5, A3-6, A3-8, A3-9, B1, B10, B11, B12, B13, B14, B15, B16, B17, B18, B19, B2, B3, B4, B5, B6, B7, B8, B9, B25, B26, B27, B28, B29, B30, B31, B32, B33, B34, B35, B36, B37, B38, B39, B40, B41, B42, EN-10, EN-11, EN-3, EN-4, EN-5, EN-6, EN-9

Figure 10

Row labels (100 FCs, bottom to top)

26, 41, 53, 10, 103, 63, 60, 55, 2, 4, 110, 77, 33, 88, 7, 39, 31, 50, 96, 24, 8, 13, 9, 57, 30, 34, 3, 35, 75, 104, 70, 59, 102, 44, 138, 5, 126, 84, 47, 116, 23, 29, 56, 32, 92, 65, 72, 94, 85, 42, 38, 12, 123, 139, 131, 137, 40, 90, 62, 122, 71, 51, 25, 48, 113, 105, 101, 118, 52, 74, 124, 81, 17, 86, 87, 79, 54, 6, 130, 99, 66, 98, 58, 111, 100, 108, 109, 114, 127, 107, 132, 95, 80, 115, 120, 78, 19, 49, 125, 89

Table S1: MeSH Annotations of Representative Compendium Samples

Mesh Categories	Counts
**Anatomy**	
Cells [A11]	1597
Tissues [A10]	1051
Hemic and Immune Systems [A15]	701
Body Regions [A01]	242
Digestive System [A03]	182
Urogenital System [A05]	95
Musculoskeletal System [A02]	94
Respiratory System [A04]	85
Nervous System [A08]	64
Endocrine System [A06]	50
Embryonic Structures [A16]	50
Integumentary System [A17]	42
Cardiovascular System [A07]	23
Fluids and Secretions [A12]	13
Stomatognathic System [A14]	12
Animal Structures [A13]	10
Sense Organs [A09]	8
Plant Structures [A18]	1
Viral Structures [A21]	1
Fungal Structures [A19]	0
Bacterial Structures [A20]	0
**Diseases**	
Neoplasms [C04]	1030
Pathological Conditions, Signs and Symptoms [C23]	585
Skin and Connective Tissue Diseases [C17]	194
Immune System Diseases [C20]	192
Female Urogenital Diseases and Pregnancy Complications [C13]	126
Respiratory Tract Diseases [C08]	70
Bacterial Infections and Mycoses [C01]	62
Nervous System Diseases [C10]	40
Endocrine System Diseases [C19]	31
Hemic and Lymphatic Diseases [C15]	29
Wounds and Injuries [C26]	21
Virus Diseases [C02]	20
Male Urogenital Diseases [C12]	20
Digestive System Diseases [C06]	12
Cardiovascular Diseases [C14]	11

Nutritional and Metabolic Diseases [C18]	11
Eye Diseases [C11]	10
Congenital, Hereditary, and Neonatal Diseases and Abnormalities [C16]	6
Musculoskeletal Diseases [C05]	3
Stomatognathic Diseases [C07]	2
Parasitic Diseases [C03]	1
Animal Diseases [C22]	1
Otorhinolaryngologic Diseases [C09]	0
Disorders of Environmental Origin [C21]	0
Occupational Diseases [C24]	0
Chemically-Induced Disorders [C25]	0

Chemicals and Drugs

Nucleic Acids, Nucleotides, and Nucleosides [D13]	521
Organic Chemicals [D02]	261
Heterocyclic Compounds [D03]	186
Inorganic Chemicals [D01]	150
Amino Acids, Peptides, and Proteins [D12]	134
Carbohydrates [D09]	106
Polycyclic Compounds [D04]	99
Biological Factors [D23]	97
Chemical Actions and Uses [D27]	80
Enzymes and Coenzymes [D08]	56
Hormones, Hormone Substitutes, and Hormone Antagonists [D06]	52
Pharmaceutical Preparations [D26]	18
Lipids [D10]	15
Complex Mixtures [D20]	14
Macromolecular Substances [D05]	13
Biomedical and Dental Materials [D25]	0

Phenomena and Processes

Genetic Phenomena [G05]	1188
Physical Phenomena [G01]	328
Reproductive and Urinary Physiological Phenomena [G08]	178
Physiological Phenomena [G07]	138
Biological Phenomena [G16]	70
Mathematical Concepts [G17]	61
Cell Physiological Phenomena [G04]	22
Chemical Phenomena [G02]	11
Immune System Phenomena [G12]	4
Microbiological Phenomena [G06]	2
Circulatory and Respiratory Physiological Phenomena [G09]	2
Metabolic Phenomena [G03]	1

Musculoskeletal and Neural Physiological Phenomena [G11]	1
Plant Physiological Phenomena [G15]	1
Digestive System and Oral Physiological Phenomena [G10]	0
Integumentary System Physiological Phenomena [G13]	0
Ocular Physiological Phenomena [G14]	0

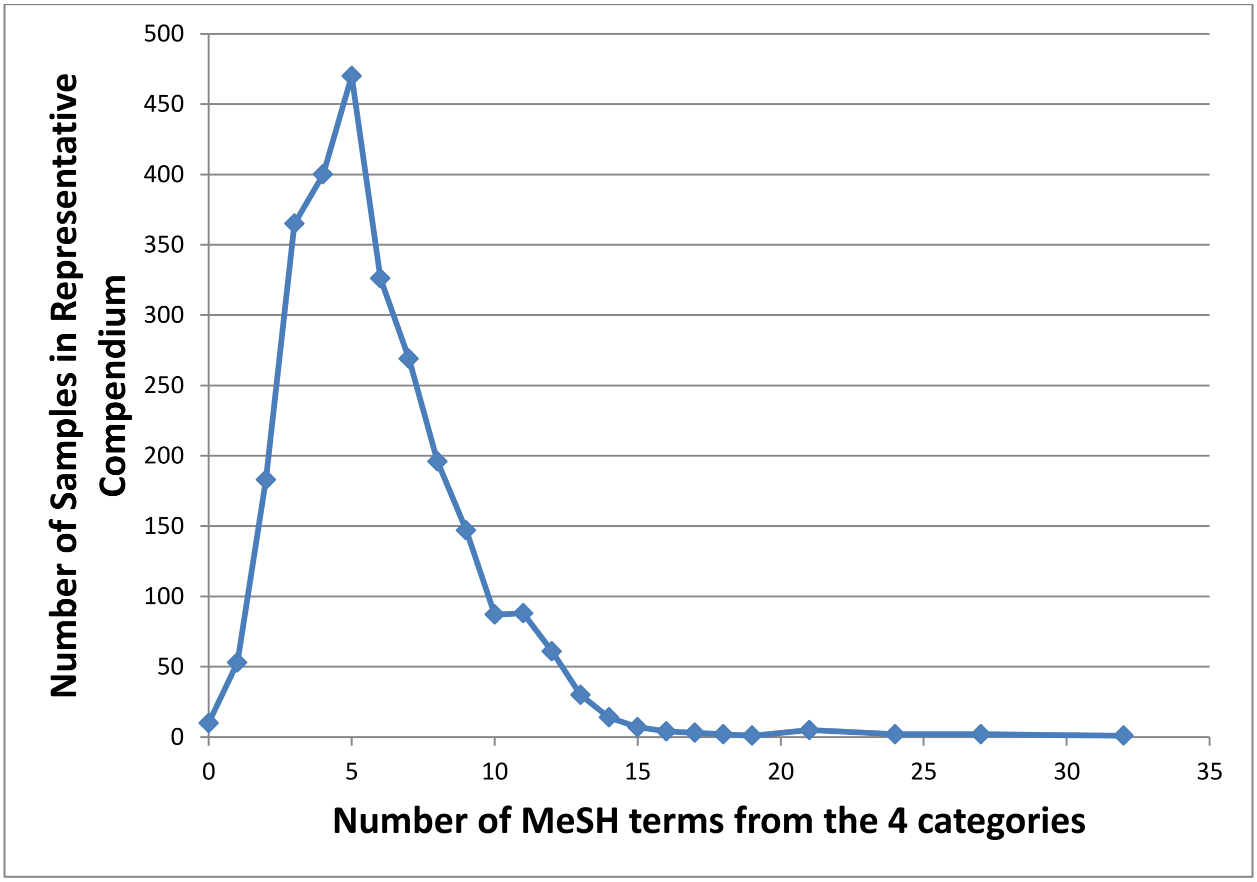

Figure S1: KNN-kneeplots from Full Compendium

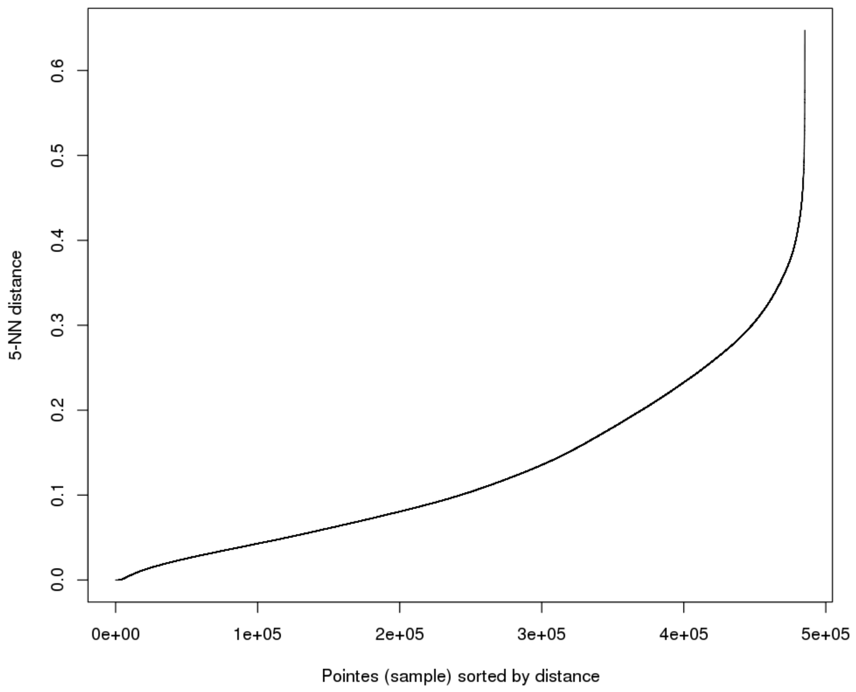

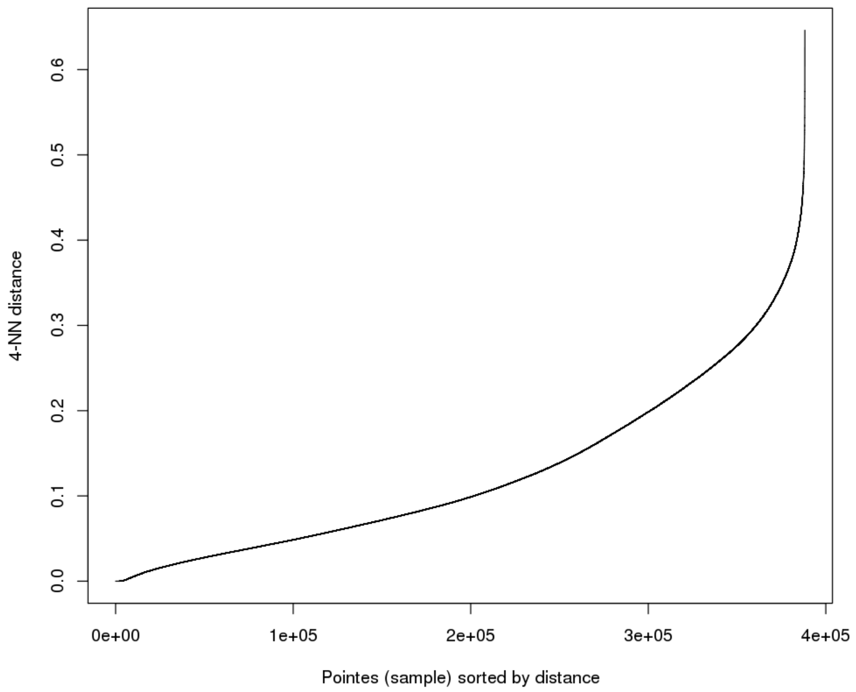

Figure S2: Tanglegram of FC and Gene space dendrograms (MAQC data)

High resolution image is available as a separate PDF file in supplementary (FigureS2_highres.pdf).

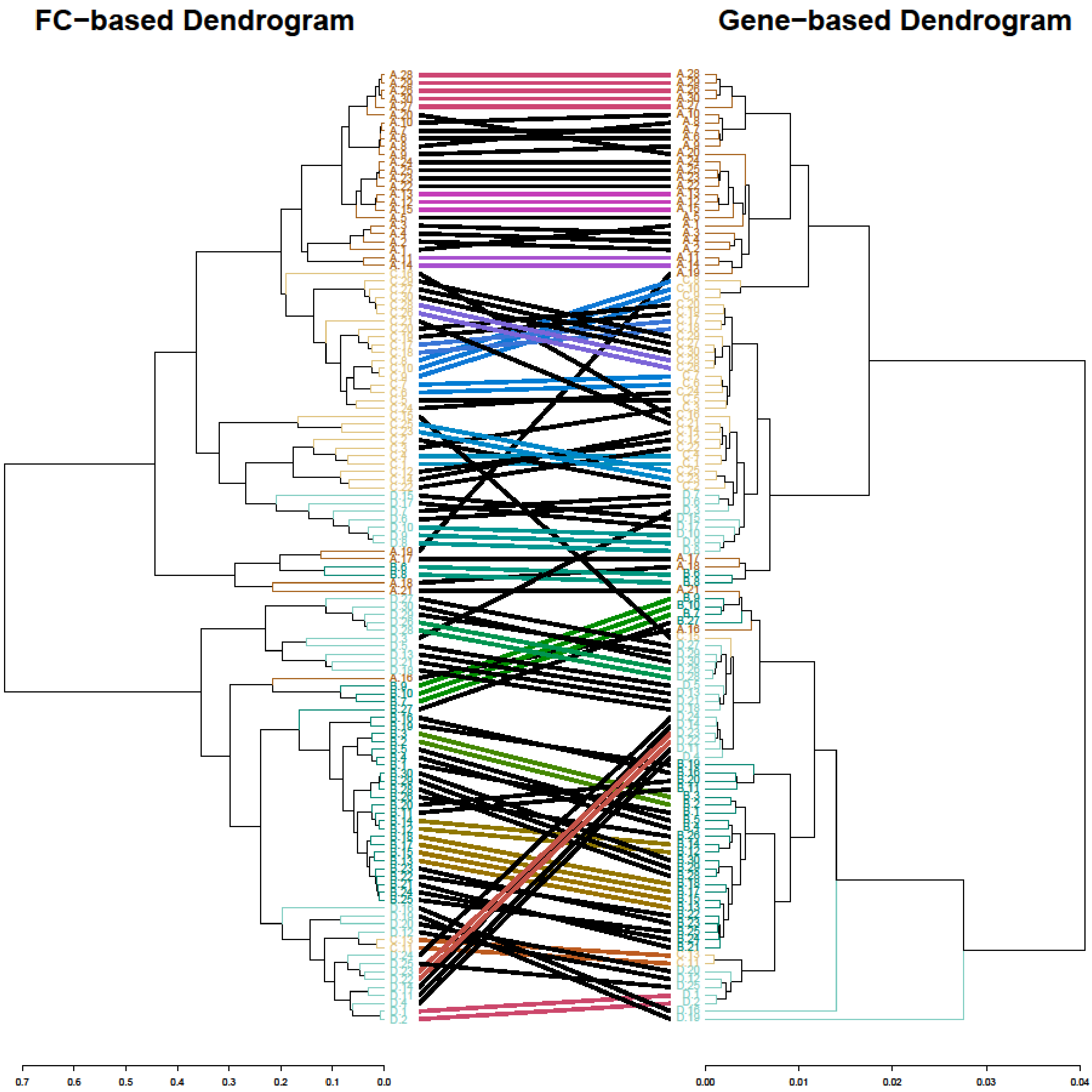

Figure S3: Heatmap of all samples in nervous system (GSE3536)

High resolution image is available as a separate PDF file in supplementary (FigureS3_highres.pdf).

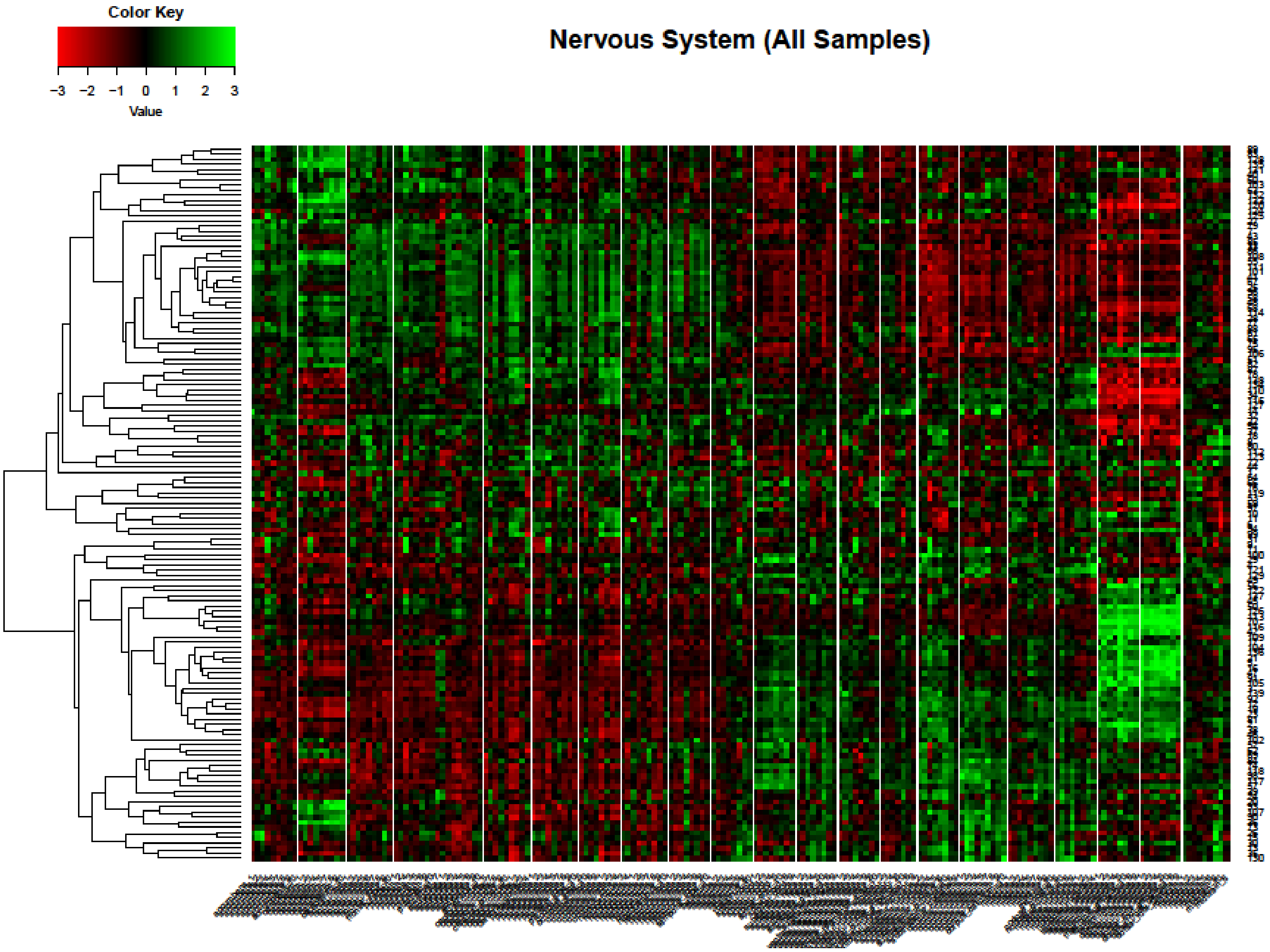

Figure S4: Clustering of GSE71370 samples using 6636 DE genes

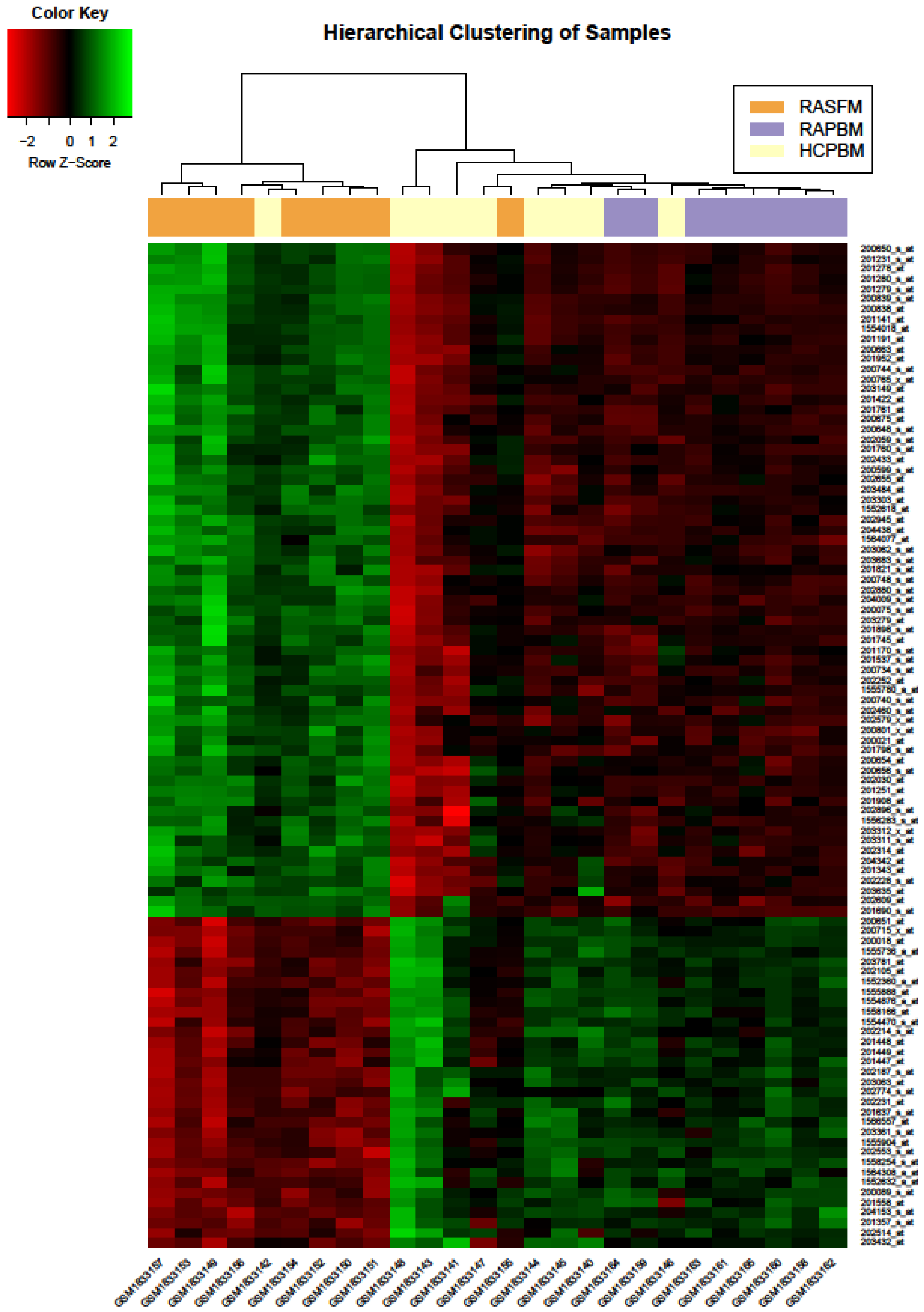

Figure S5: Reanalysis of GSE7538 (Parthenolide study used by Engreitz et al)

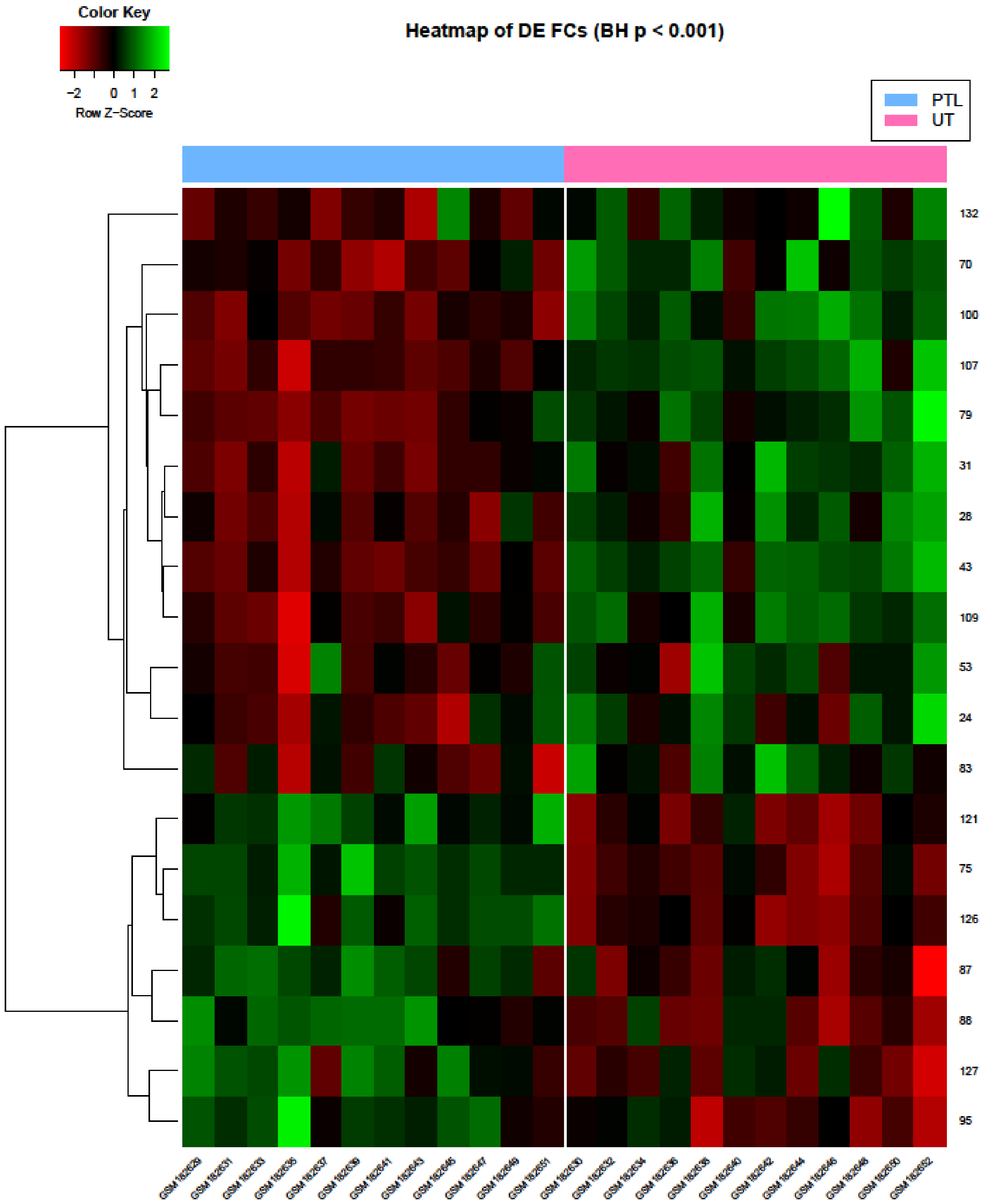

Figure S6: Number of unique GO terms versus number of leading principal components from PCA

Similar to how GO annotations were obtained for the FCs, we obtained GO annotations for the principal components (based on PCA on the same representative compendium). The cumulative number of GO unique codes can be considered as an indicator of the biological relevancy of each sequential component.

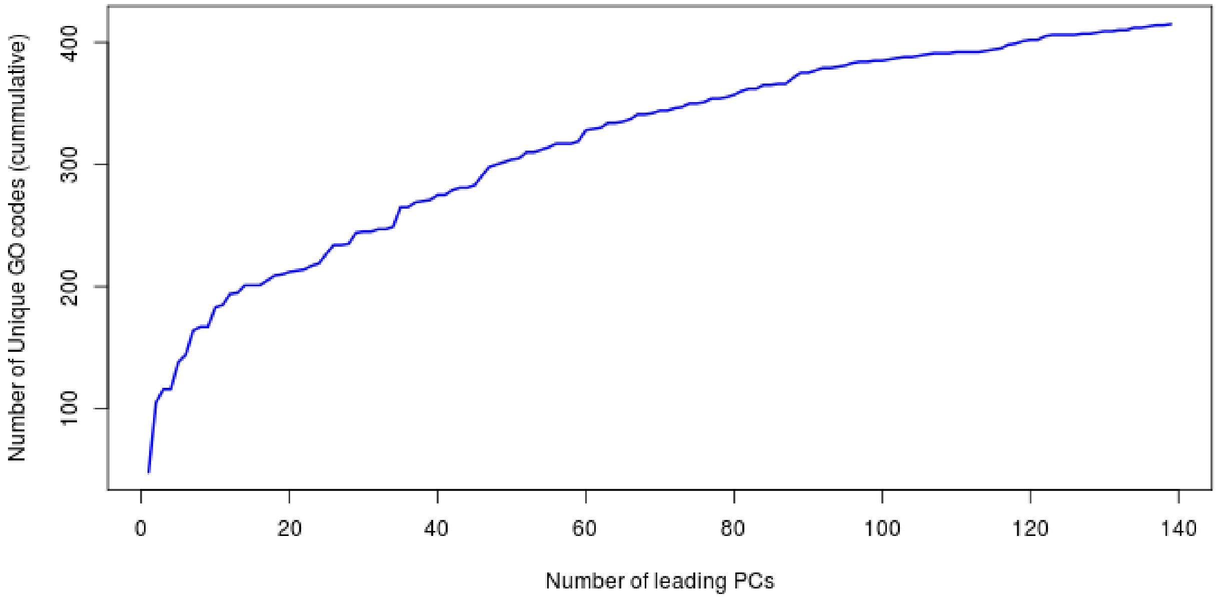

The plateau in the number of unique GO codes at around PC 139 suggests that the number of PC-based components to be retained for the current input data were within the neighborhood.

Table S2: Full GO annotations for DE FCs in E-MTAB-3162

FC	BH-Corrected P-value	GO ID	Description
2	0.00933	GO:0045071	negative regulation of viral genome repl...
		GO:0002479	antigen processing and presentation of e...
		GO:0035455	response to interferon-alpha
		GO:0002480	antigen processing and presentation of e...
		GO:0032480	negative regulation of type I interferon...
		GO:0035456	response to interferon-beta
		GO:0034341	response to interferon-gamma
		GO:0019885	antigen processing and presentation of e...
		GO:0016032	viral process
		GO:0006955	immune response
		GO:0032020	ISG15-protein conjugation
		GO:0035458	cellular response to interferon-beta
		GO:0043123	positive regulation of I-kappaB kinase/N...
		GO:0046597	negative regulation of viral entry into ...
		GO:0050852	T cell receptor signaling pathway
		GO:0042270	protection from natural killer cell medi...
		GO:0019060	intracellular transport of viral protein...
		GO:0009597	detection of virus
		GO:1902187	negative regulation of viral release fro...
		GO:0045089	positive regulation of innate immune res...
		GO:0033209	tumor necrosis factor-mediated signaling...
		GO:0045824	negative regulation of innate immune res...
		GO:0019941	modification-dependent protein catabolic...
		GO:0042102	positive regulation of T cell proliferat...
4	0.00933	GO:0007067	mitotic nuclear division
		GO:0000278	mitotic cell cycle
		GO:0000082	G1/S transition of mitotic cell cycle
		GO:0007264	small GTPase mediated signal transductio...
		GO:0034080	CENP-A containing nucleosome assembly
		GO:0006271	DNA strand elongation involved in DNA re...
		GO:0006270	DNA replication initiation
		GO:0000086	G2/M transition of mitotic cell cycle
		GO:0032201	telomere maintenance via semi-conservati...
		GO:0000722	telomere maintenance via recombination
		GO:0007059	chromosome segregation
		GO:0006260	DNA replication
		GO:0000083	regulation of transcription involved in ...
		GO:0000079	regulation of cyclin-dependent protein s...
		GO:0007076	mitotic chromosome condensation
		GO:0007080	mitotic metaphase plate congression
		GO:0045003	double-strand break repair via synthesis...
		GO:0008283	cell proliferation

		GO:0000281	mitotic cytokinesis
		GO:0000724	double-strand break repair via homologou...
		GO:0006284	base-excision repair
		GO:0051726	regulation of cell cycle
		GO:0071897	DNA biosynthetic process
		GO:0051988	regulation of attachment of spindle micr...
		GO:0032508	DNA duplex unwinding
		GO:0032467	positive regulation of cytokinesis
		GO:0000076	DNA replication checkpoint
		GO:0010032	meiotic chromosome condensation
		GO:0000727	double-strand break repair via break-ind...
		GO:0006297	nucleotide-excision repair, DNA gap fill...
		GO:0007018	microtubule-based movement
		GO:0034501	protein localization to kinetochore
		GO:0000070	mitotic sister chromatid segregation
		GO:0042769	DNA damage response, detection of DNA da...
		GO:0007088	regulation of mitotic nuclear division
		GO:0007094	mitotic spindle assembly checkpoint
		GO:0042276	error-prone translesion synthesis
		GO:0051382	kinetochore assembly
		GO:0007052	mitotic spindle organization
		GO:0051256	mitotic spindle midzone assembly
		GO:0006298	mismatch repair
		GO:0031577	spindle checkpoint
		GO:0090307	mitotic spindle assembly
		GO:0051321	meiotic cell cycle
		GO:0046602	regulation of mitotic centrosome separat...
		GO:0085020	protein K6-linked ubiquitination
		GO:0001556	oocyte maturation
		GO:0007019	microtubule depolymerization
		GO:0070987	error-free translesion synthesis
		GO:0006268	DNA unwinding involved in DNA replicatio...
		GO:0043137	DNA replication, removal of RNA primer
		GO:0019886	antigen processing and presentation of e...
		GO:0033683	nucleotide-excision repair, DNA incision
		GO:0031110	regulation of microtubule polymerization...
		GO:0051488	activation of anaphase-promoting complex...
		GO:0060236	regulation of mitotic spindle organizati...
		GO:0048478	replication fork protection
		GO:0031145	anaphase-promoting complex-dependent pro...
12	0.00933	GO:0006954	inflammatory response
		GO:0030593	neutrophil chemotaxis
		GO:0045087	innate immune response
		GO:0031295	T cell costimulation
		GO:0002250	adaptive immune response
		GO:0019886	antigen processing and presentation of e...

		GO:0060333	interferon-gamma-mediated signaling path...
		GO:0006898	receptor-mediated endocytosis
		GO:0050776	regulation of immune response
		GO:0070374	positive regulation of ERK1 and ERK2 cas...
		GO:0050852	T cell receptor signaling pathway
		GO:0006955	immune response
		GO:0050729	positive regulation of inflammatory resp...
		GO:0030168	platelet activation
		GO:0035589	G-protein coupled purinergic nucleotide ...
		GO:0050853	B cell receptor signaling pathway
		GO:0042102	positive regulation of T cell proliferat...
		GO:0007229	integrin-mediated signaling pathway
		GO:0050715	positive regulation of cytokine secretio...
		GO:0006958	complement activation, classical pathway
		GO:0006968	cellular defense response
		GO:0007596	blood coagulation
		GO:0007169	transmembrane receptor protein tyrosine ...
		GO:0071222	cellular response to lipopolysaccharide
		GO:0045579	positive regulation of B cell differenti...
		GO:0042742	defense response to bacterium
		GO:0071727	cellular response to triacyl bacterial l...
		GO:0002503	peptide antigen assembly with MHC class ...
		GO:0001816	cytokine production
		GO:0032729	positive regulation of interferon-gamma ...
		GO:0007155	cell adhesion
		GO:0006935	Chemotaxis
		GO:0006911	phagocytosis, engulfment
		GO:0045730	respiratory burst
		GO:0032760	positive regulation of tumor necrosis fa...
		GO:0016064	immunoglobulin mediated immune response
		GO:0043306	positive regulation of mast cell degranu...
		GO:0050766	positive regulation of phagocytosis
		GO:0019371	cyclooxygenase pathway
		GO:0040037	negative regulation of fibroblast growth...
		GO:0048661	positive regulation of smooth muscle cel...
		GO:0002690	positive regulation of leukocyte chemota...
19	0.00933	GO:0006954	inflammatory response
		GO:0002576	platelet degranulation
		GO:0042742	defense response to bacterium
		GO:0032496	response to lipopolysaccharide
		GO:0008360	regulation of cell shape
		GO:0002755	MyD88-dependent toll-like receptor signa...
		GO:0050776	regulation of immune response
		GO:0006955	immune response
		GO:0006968	cellular defense response
		GO:0050729	positive regulation of inflammatory resp...

		GO:0038094	Fc-gamma receptor signaling pathway
		GO:0002752	cell surface pattern recognition recepto...
		GO:0010575	positive regulation of vascular endothel...
		GO:0045429	positive regulation of nitric oxide bios...
		GO:0045087	innate immune response
		GO:0030168	platelet activation
		GO:0030593	neutrophil chemotaxis
		GO:0007155	cell adhesion
		GO:0050707	regulation of cytokine secretion
		GO:0042535	positive regulation of tumor necrosis fa...
		GO:0030041	actin filament polymerization
		GO:0006952	defense response
		GO:0002250	adaptive immune response
		GO:0045766	positive regulation of angiogenesis
		GO:0071222	cellular response to lipopolysaccharide
		GO:0038123	toll-like receptor TLR1:TLR2 signaling p...
		GO:0038124	toll-like receptor TLR6:TLR2 signaling p...
		GO:0002374	cytokine secretion involved in immune re...
		GO:0071727	cellular response to triacyl bacterial l...
		GO:0050702	interleukin-1 beta secretion
		GO:0032755	positive regulation of interleukin-6 pro...
		GO:0007166	cell surface receptor signaling pathway
		GO:0006935	Chemotaxis
		GO:0051603	proteolysis involved in cellular protein...
38	0.00933	GO:0006958	complement activation, classical pathway
		GO:0070098	chemokine-mediated signaling pathway
		GO:0022617	extracellular matrix disassembly
		GO:0007267	cell-cell signaling
		GO:0030574	collagen catabolic process
		GO:0006955	immune response
		GO:0010951	negative regulation of endopeptidase act...
		GO:0007155	cell adhesion
		GO:0006954	inflammatory response
		GO:0006955	immune response
		GO:0045060	negative thymic T cell selection
		GO:0031295	T cell costimulation
82	0.00933	GO:0045669	positive regulation of osteoblast differ...
		GO:0003094	glomerular filtration
		GO:0035456	response to interferon-beta
		GO:0051607	defense response to virus
		GO:0060337	type I interferon signaling pathway

Table S3: Full GO annotations for DE FCs in GSE71370

FC	BH-Corrected P-value	GO ID	Description
105	0.0102	GO:0071294	Cellular response to zinc ion
		GO:0071276	Cellular response to cadmium ion
		GO:0044597	Daunorubicin metabolic process
		GO:0044598	Doxorubicin metabolic process
		GO:0045926	Negative regulation of growth
113	0.0102	GO:0032826	Glomerular basement membrane development
		GO:0032496	Response to lipopolysaccharide
		GO:0043542	Endothelial cell migration
48	0.0109	GO:0002250	adaptive immune response
		GO:0019886	antigen processing and presentation of e...
		GO:0031295	T cell costimulation
		GO:0045576	mast cell activation
		GO:0048007	antigen processing and presentation, exo...
		GO:0050850	positive regulation of calcium-mediated ...
		GO:0050852	T cell receptor signaling pathway
		GO:0050862	positive regulation of T cell receptor s...
		GO:0060333	interferon-gamma-mediated signaling path...
		GO:0097324	melanocyte migration
		GO:1901898	negative regulation of relaxation of car...
100	0.0109	GO:0070098	chemokine-mediated signaling pathway
		GO:0030593	neutrophil chemotaxis
		GO:0002548	monocyte chemotaxis
		GO:0071356	cellular response to tumor necrosis fact...
		GO:0015701	bicarbonate transport
81	0.0245	GO:0000122	negative regulation of transcription fro...
		GO:0001837	epithelial to mesenchymal transition
		GO:0001942	hair follicle development
		GO:0001958	endochondral ossification
		GO:0006954	inflammatory response
		GO:0006955	immune response
		GO:0007156	homophilic cell adhesion via plasma memb...
		GO:0010991	negative regulation of SMAD protein comp...
		GO:0030182	neuron differentiation
		GO:0030282	bone mineralization
		GO:0032496	response to lipopolysaccharide
		GO:0035019	somatic stem cell population maintenance
		GO:0042060	wound healing
		GO:0042127	regulation of cell proliferation
		GO:0045766	positive regulation of angiogenesis
		GO:0048013	ephrin receptor signaling pathway
		GO:0048755	branching morphogenesis of a nerve
		GO:0060021	palate development

		GO:0060325	face morphogenesis
		GO:0060394	negative regulation of pathway-restricte...
		GO:0071222	cellular response to lipopolysaccharide
		GO:0071276	cellular response to cadmium ion
		GO:0071294	cellular response to zinc ion
		GO:0071347	cellular response to interleukin-1
		GO:0071356	cellular response to tumor necrosis fact...
		GO:0090190	positive regulation of branching involve...
		GO:2000678	negative regulation of transcription reg...
		GO:2001013	epithelial cell proliferation involved i...
25	0.0284	GO:0007156	homophilic cell adhesion via plasma memb...
87	0.0284	GO:0019886	antigen processing and presentation of e...
		GO:0044130	negative regulation of growth of symbion...
		GO:0002503	peptide antigen assembly with MHC class ...
		GO:0031295	T cell costimulation
		GO:0060333	interferon-gamma-mediated signaling path...
		GO:0006955	immune response
		GO:0050832	defense response to fungus
		GO:0045416	positive regulation of interleukin-8 bio...
		GO:0001774	microglial cell activation
		GO:0006954	inflammatory response
		GO:0007155	cell adhesion
51	0.0287		
77	0.0300	GO:0000045	autophagosome assembly
		GO:0006364	rRNA processing
		GO:0006189	'de novo' IMP biosynthetic process
		GO:0030490	maturation of SSU-rRNA
		GO:0051084	'de novo' posttranslational protein fold...
		GO:0006390	transcription from mitochondrial promote...
		GO:0042254	ribosome biogenesis
		GO:0046655	folic acid metabolic process
75	0.0353	GO:1990440	positive regulation of transcription fro...
		GO:0006564	L-serine biosynthetic process
		GO:0042149	cellular response to glucose starvation
		GO:0036499	PERK-mediated unfolded protein response
		GO:0070059	intrinsic apoptotic signaling pathway in...
		GO:0002523	leukocyte migration involved in inflamma...
		GO:0030593	neutrophil chemotaxis
		GO:0070488	neutrophil aggregation
71	0.0460	GO:0031581	hemidesmosome assembly
		GO:0010951	negative regulation of endopeptidase act...
		GO:0008544	epidermis development
		GO:0022617	extracellular matrix disassembly
