# Supplementary figures and images for: Data-driven human transcriptomic modules determined by independent component analysis
Source: BMC Bioinformatics. 2018 Sep 17;19:327. doi: 10.1186/s12859-018-2338-4 (PMC6142401; doi:10.1186/s12859-018-2338-4)

FC-based Dendrogram

Gene-based Dendrogram

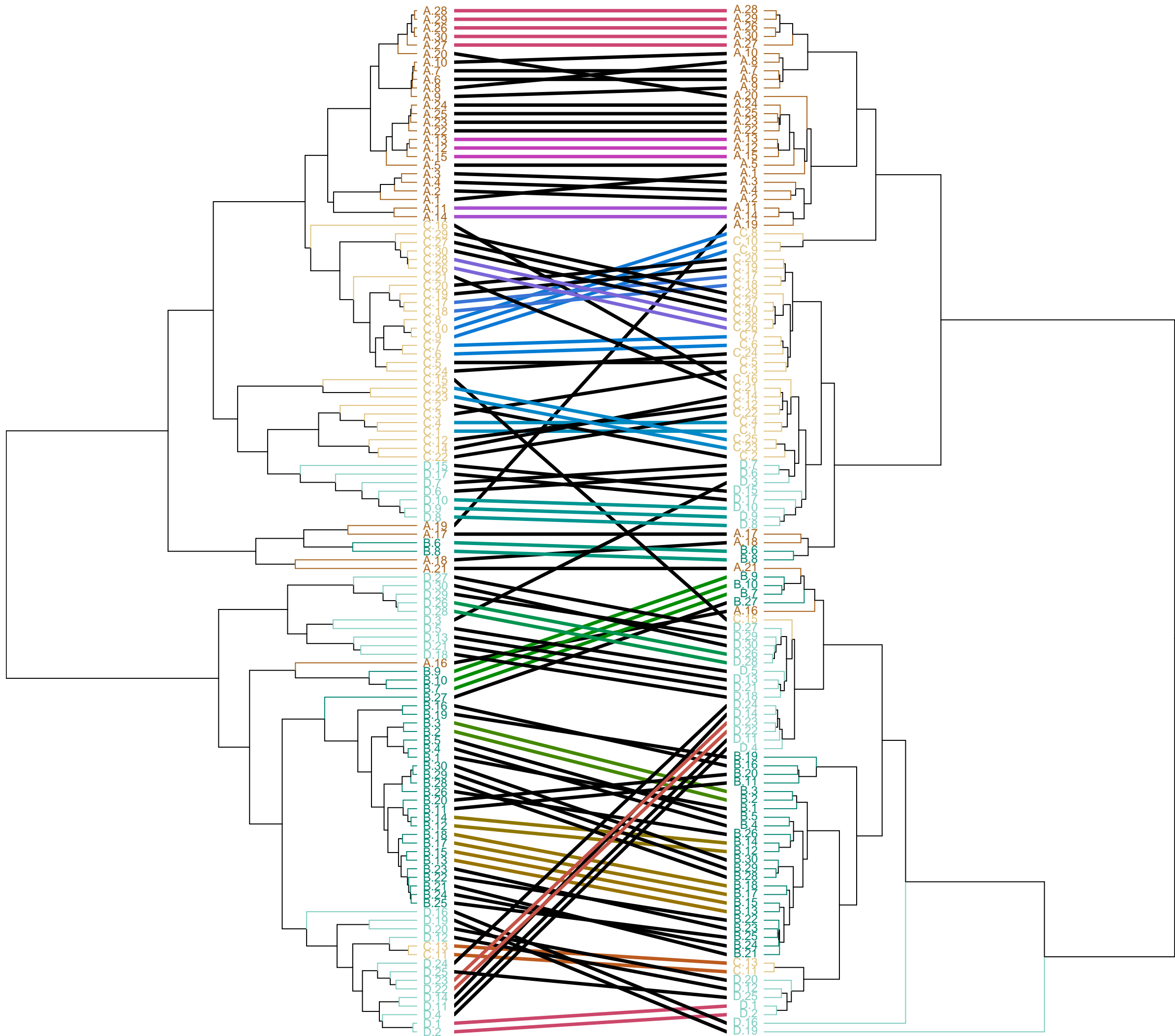

0.7 0.6 0.5 0.4 0.3 0.2 0.1 0.0

0.00 0.01 0.02 0.03 0.04

Supplement: Supplementary file 1 — Text S1. Axis labels for Figures in Paper. Table S1. MeSH Annotations of Representative Compendium Samples. Figure S1. KNN-kneeplots from Full Compendium. Figure S2. Tanglegram of FC and Gene space dendrograms (MAQC data). Figure S3. Heatmap of all samples in nervous system (GSE3536). Figure S4. Clustering of GSE71370 samples using 6636 DE genes. Figure S5. Reanalysis of GSE7538 (Parthenolide study used by Engreitz et al.). Figure S6. Number of unique GO terms versus number of leading principal components from PCA. Table S2. Full GO annotations for DE FCs in E-MTAB-3162. Table S3. Full GO annotations for DE FCs in GSE71370. (ZIP 1335 kb) [file 12859_2018_2338_MOESM1_ESM.zip › FigureS2_highres.pdf]

# Nervous System (All Samples)

Color Key

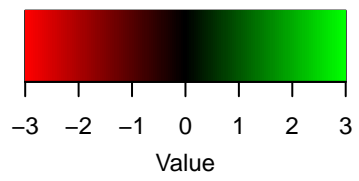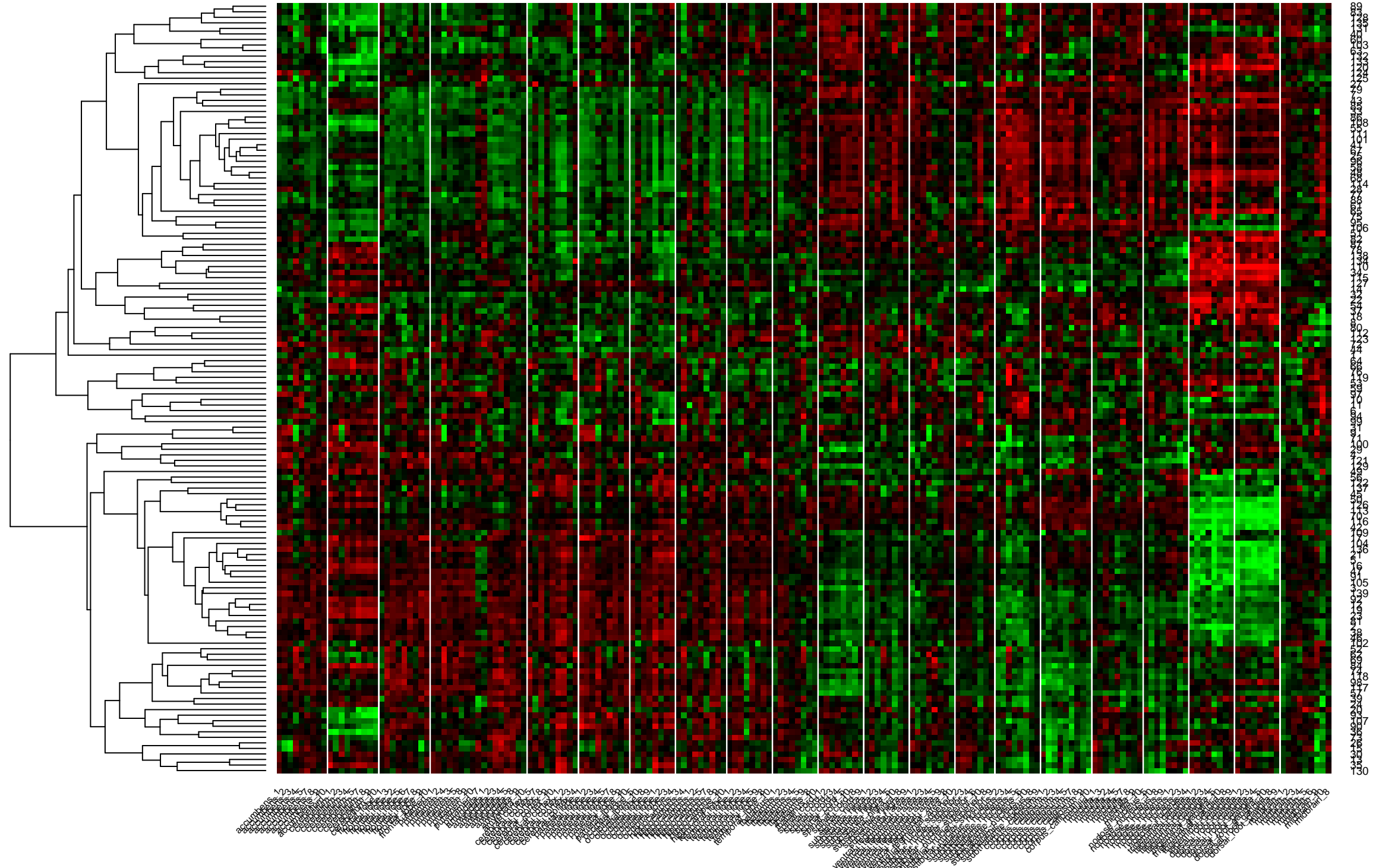

Supplement: Supplementary file 1 — Text S1. Axis labels for Figures in Paper. Table S1. MeSH Annotations of Representative Compendium Samples. Figure S1. KNN-kneeplots from Full Compendium. Figure S2. Tanglegram of FC and Gene space dendrograms (MAQC data). Figure S3. Heatmap of all samples in nervous system (GSE3536). Figure S4. Clustering of GSE71370 samples using 6636 DE genes. Figure S5. Reanalysis of GSE7538 (Parthenolide study used by Engreitz et al.). Figure S6. Number of unique GO terms versus number of leading principal components from PCA. Table S2. Full GO annotations for DE FCs in E-MTAB-3162. Table S3. Full GO annotations for DE FCs in GSE71370. (ZIP 1335 kb) [file 12859_2018_2338_MOESM1_ESM.zip › FigureS3_highres.pdf]
